# Initiators of Classical and Lectin Complement Pathways Are Differently Engaged after Traumatic Brain Injury—Time-Dependent Changes in the Cortex, Striatum, Thalamus and Hippocampus in a Mouse Model

**DOI:** 10.3390/ijms22010045

**Published:** 2020-12-22

**Authors:** Agata Ciechanowska, Katarzyna Ciapała, Katarzyna Pawlik, Marco Oggioni, Domenico Mercurio, Maria-Grazia De Simoni, Joanna Mika

**Affiliations:** 1 Department of Pain Pharmacology, Maj Institute of Pharmacology, Polish Academy of Sciences, 31-343 Krakow, Poland; ciechan@if-pan.krakow.pl (A.C.); kat.ciapala@gmail.com (K.C.); pawlik@if-pan.krakow.pl (K.P.); 2 Department of Neuroscience, Istituto di Ricerche Farmacologiche Mario Negri-IRCCS, 20156 Milan, Italy; marco.oggioni@marionegri.it (M.O.); domenico.mercurio@marionegri.it (D.M.); mariagrazia.desimoni@marionegri.it (M.-G.D.S.)

**Keywords:** complement component 1q (*C1q*), complement component 1s (*C1s*), complement component 1r (*C1r*), collectin 11, ficolin A, ficolin B, mannose binding lectin A (MBL-A), mannose binding lectin C (MBL-C), cortex, striatum, thalamus, hippocampus

## Abstract

The complement system is involved in promoting secondary injury after traumatic brain injury (TBI), but the roles of the classical and lectin pathways leading to complement activation need to be clarified. To this end, we aimed to determine the ability of the brain to activate the synthesis of classical and lectin pathway initiators in response to TBI and to examine their expression in primary microglial cell cultures. We have modeled TBI in mice by controlled cortical impact (CCI), a clinically relevant experimental model. Using Real-time quantitative polymerase chain reaction (RT-qPCR) we analyzed the expression of initiators of classical the complement component 1q, 1r and 1s (*C1q*, *C1r*, and *C1s*) and lectin (mannose binding lectin A, mannose binding lectin C, *collectin 11*, *ficolin A*, and *ficolin B*) complement pathways and other cellular markers in four brain areas (cortex, striatum, thalamus and hippocampus) of mice exposed to CCI from 24 h and up to 5 weeks. In all murine ipsilateral brain structures assessed, we detected long-lasting, time- and area-dependent significant increases in the mRNA levels of all classical (*C1q*, *C1s*, *C1r*) and some lectin (*collectin 11, ficolin A, ficolin B*) initiator molecules after TBI. In parallel, we observed significantly enhanced expression of cellular markers for neutrophils (*Cd177*), T cells (*Cd8*), astrocytes (glial fibrillary acidic protein—*GFAP*), microglia/macrophages (allograft inflammatory factor 1—*IBA-1*), and microglia (transmembrane protein 119—*TMEM119*); moreover, we detected astrocytes (GFAP) and microglia/macrophages (IBA-1) protein level strong upregulation in all analyzed brain areas. Further, the results obtained in primary microglial cell cultures suggested that these cells may be largely responsible for the biosynthesis of classical pathway initiators. However, microglia are unlikely to be responsible for the production of the lectin pathway initiators. Immunofluorescence analysis confirmed that at the site of brain injury, the C1q is localized in microglia/macrophages and neurons but not in astroglial cells. In sum, the brain strongly reacts to TBI by activating the local synthesis of classical and lectin complement pathway activators. Thus, the brain responds to TBI with a strong, widespread and persistent upregulation of complement components, the targeting of which may provide protection in TBI.

## 1. Introduction

Traumatic brain injury (TBI) persists as a leading cause of disability and mortality affecting the worldwide community. TBI is known to be associated with primary mechanical damage that results in secondary pathogenic cellular cascades, activated within minutes following injury, that interacts in a complex network, which leads to cell death or recovery [1]. In patients surviving TBI, this secondary injury cascade, which is closely related to activation of the inflammatory response, is believed to be responsible for the evolution of the brain damage [2,3]. Not enough is known about the cellular and molecular changes occurring during the secondary damage, which evolves over days after the injury. Gaining knowledge about these changes will be very important for development of novel pharmacological and therapeutic interventions [4]. 

The complement system is a powerful effector tool of innate and adaptive immunity. In the brain, the complement system is known to subserve multiple functions, including regulation of synaptic pruning during development, control of homeostatic functions in the uninjured brain, and coordination of the inflammatory response following injury. There are three pathways leading to full activation of the complement cascade: the classical, the alternative, and the lectin pathway, each responding to specific danger signals and characterized by specific initiators and enzymes. The classical pathway is activated following the binding of the C1q-C1s-C1r complex to antigen-antibody complexes or directly to specific molecules (i.e., beta-amyloid, C reactive protein, DNA, and apoptotic bodies). The lectin pathway activator molecules include different pattern recognition molecules, such as mannose binding lectins (MBLs, found in rodents in two isoforms: A and C) and collectin-11, or by ficolins (found in rodents in two isoforms: A and B), which bind to high-density arrays of mannose, fucose, and N-acetylated sugars present on the surface of injured cells or pathogens. Spontaneous hydrolysis of circulating C3 (tick-over process) into C3 (H_2_O) on cellular surfaces acts as activator of the alternative pathway. The three pathways converge into a common pathway, resulting in the formation of the terminal C5b-9 complement complex the effector of cell lysis. A few fragments which are produced along the cascade, such as C3b, C4b, and C5b, possess direct inflammatory properties and act as opsonins, inducing an overactivation of the phagocytic response. In addition, the complement components coordinate an innate and adaptive immune reaction by interacting with multiple immune cells [5]. Complement activation results in the production of anaphylatoxins (C3a and C5a), powerful mediators of inflammation, which causes recruitment and activation of different cells, including neutrophils, T cells and macrophages. Neutrophils, which are the most abundant leukocytes in circulation, are also the first-line immune cells that reach the sites of injury [6,7]. Brain Cd8+ T cells may impact prolonged motor deficits and myelin pathology. Moreover, TBI-related neuroinflammation is also characterized by changes in the morphology and number of oligodendrocytes, astrocytes and microglia/macrophages [6,7,8,9]. Recently, clinical studies have supported these data, showing the maintenance of sustained astrocytes and microglial activation in the brains of TBI patients [10,11]. 

The complement system contributes to secondary injury in TBI [5,12], but while it is well established that injury activates the complement cascade as a systemic response and that circulating complement components get into the brain through the injured blood-brain barrier (BBB), little is known about local activation. Therefore, the purpose of our research was to determine the ability of the brain to activate the synthesis of classical and lectin pathway initiators in response to TBI and to examine their expression in different types of brain cells. Based on previous experiments, we performed controlled cortical impact (CCI) in mice, which is a clinically relevant experimental model of TBI that causes a large inflammatory response and drives the progression of brain injury [3,13,14]. We examined concomitant spatiotemporal changes occurring at the mRNA and/or protein levels of classical (C1q, C1s, and C1r) and lectin (MBL-A, MBL-C, collectin 11, ficolin A, and ficolin B) complement factors as well as cellular markers of neutrophils (Cd177), T cells (Cd8), oligodendrocytes (oligodendrocyte transcription factor 2—Olig2), astrocytes (GFAP), microglia/macrophages (IBA-1), and microglia (TMEM119) in various brain structures (cortex, striatum, thalamus, and hippocampus) after TBI. Moreover, we used confocal microscopy to study the C1q cellular localization in the contused cortex after TBI. We also studied the effect of lipopolysaccharide (LPS), an inflammatory stimulus, on the levels of a few classical and lectin pathway components in primary microglial cell cultures. 

## 2. Results

### 2.1. Time-Dependent Study of Cd177, Cd8 and Olig2 mRNA Expression in the Cortex, Striatum, Thalamus, and Hippocampus after TBI in Mice

**Figure 1:**
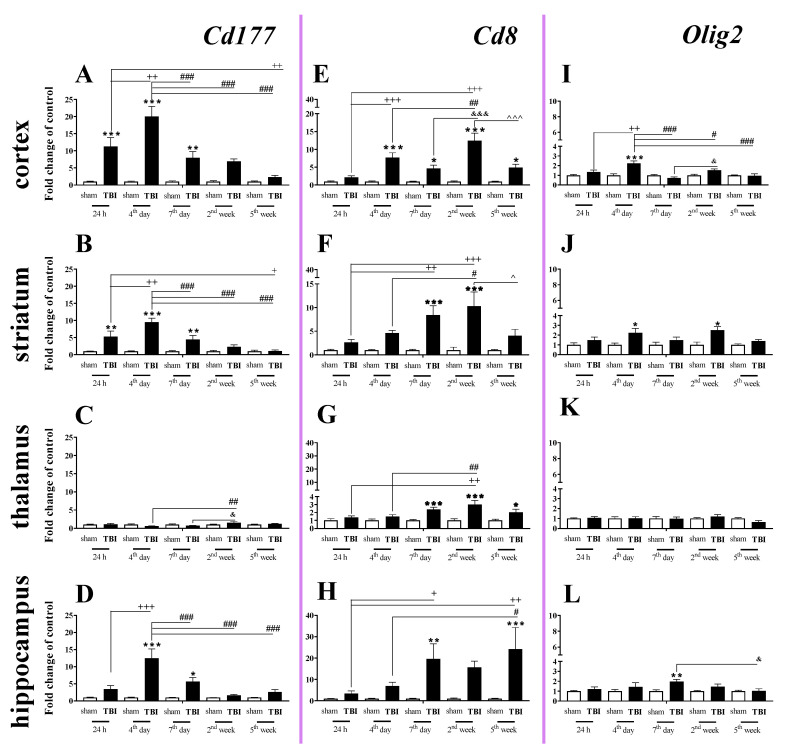
(**A**–**L**). The mRNA expression changes of *Cd177*, *Cd8*, and *Olig2* in the cortex, striatum, thalamus, and hippocampus of TBI compared to sham-injured animals at different time points (24 h–5 weeks). The data are given as fold changes relative to the control as mean ± SEM (standard error of the mean) (sham groups, *n* = 5–8; TBI groups, *n* = 4–8). * *p* < 0.05; ** *p* < 0.01; *** *p* < 0.001 show differences considered statistically significant between the sham and TBI groups, + *p* < 0.05; ++ *p* < 0.01; +++ *p* < 0.001 show differences considered statistically significant comparing to the 24 h TBI group; # *p* < 0.05; ## *p* < 0.01; ### *p* < 0.001 show differences considered statistically significant comparing to the 4th day TBI group; & *p* < 0.05; &&& *p* < 0.001 show differences considered statistically significant when comparing to the 7th day TBI group; ^ *p* < 0.05; ^^^ *p* < 0.001 show differences considered statistically significant comparing to the 2 weeks TBI group as estimated by two-way ANOVA followed by Bonferroni’s post hoc test.

The elevated mRNA expression of *Cd177* (a cellular marker of neutrophils) was observed in the cortex, striatum, and hippocampus (Figure 1A,B,D). In the cortex and striatum, *Cd177* upregulation began 24 h after TBI, was the highest on the 4th day and remained elevated until the 7th day after traumatic brain injury when compared to that of sham-operated animals (Figure 1A,B).

In the hippocampus, *Cd177* upregulation began and reached a peak 4 days after brain injury and then decreased after day 7. We did not observe any changes in expression of *Cd177* in the thalamus (Figure 1C). For the mRNA expression of *Cd177*, the two-way ANOVA confirmed a significant interaction between the considered time points and TBI for the cortex (F_4,55_ = 9.34; *p* < 0.0001) (Figure 1A), striatum (F_4,55_ = 7.88; *p* < 0.0001) (Figure 1B), and hippocampus (F_4,52_ = 7.47; *p* < 0.0001) (Figure 1D).

Upregulation of *Cd8* mRNA expression (a cellular marker of T cells) was observed in all studied structures. The changes began in the cortex 4 days after injury and in the striatum and hippocampus on the 7th day. When comparing to the mRNA level of sham-operated animals, the highest value was observed 2 weeks after brain injury in cortex and striatum and 5 weeks after injury in the hippocampus. In the cortex and hippocampus, *Cd8* mRNA remained elevated until the 5th week, which was the last time point (Figure 1E,H). For the mRNA expression of *Cd8*, the two-way ANOVA confirmed a significant interaction between the considered time points and TBI in the cortex (F_4,55_ = 8.76; *p* < 0.0001) (Figure 1E), striatum (F_4,55_ = 3.52; *p* = 0.0125) (Figure 1F), and hippocampus (F_4,53_ = 2.76; *p* = 0.0372) (Figure 1H). Moreover, after TBI, we observed upregulation of *Cd8* in the thalamus (Figure 1G) between the 7th day and 5th week when compared to that in sham animals, according to Bonferroni’s post hoc analysis.

The expression of *Olig2* (a marker of oligodendrocytes) was significantly elevated in the cortex, and the upregulation was observed 4 days after injury. For the mRNA expression of *Olig2* in the cortex, two-way ANOVA confirmed a significant interaction between the considered time points and TBI (F_4,54_ = 7.06; *p* = 0.0001) (Figure 1I). Moreover, we observed upregulation of *Olig2* in the striatum (Figure 1J) on the 4th day and 2nd week and hippocampus on the day 7th after TBI when compared to that in sham animals, according to Bonferroni’s post hoc analysis. No changes in mRNA expression were obtained in the thalamus.

### 2.2. Time-Dependent Study of GFAP, IBA-1 and TMEM119 mRNA Expression in the Cortex, Striatum, Thalamus, and Hippocampus after TBI in Mice

**Figure 2:**
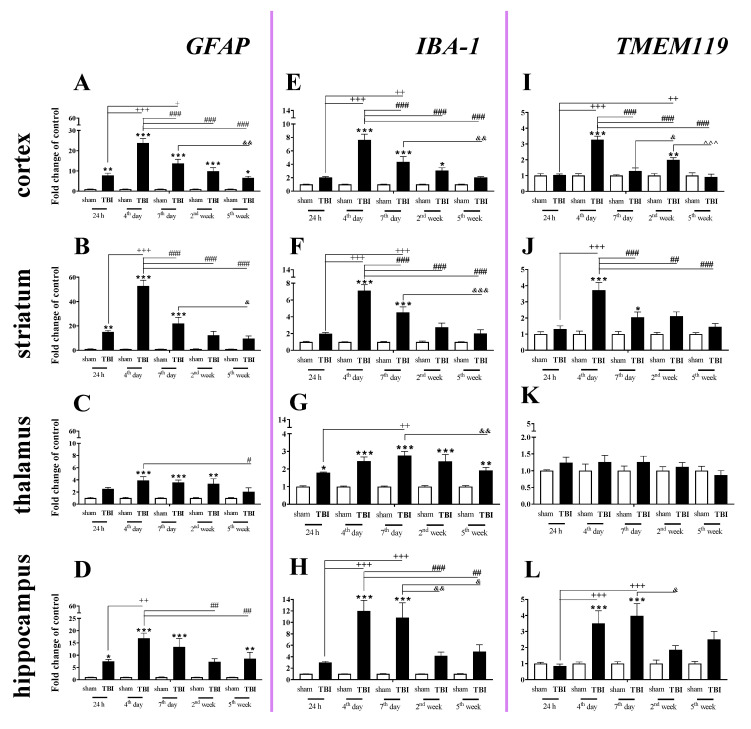
(**A**–**L**). The mRNA expression changes of *GFAP, IBA-1*, and *TMEM119* in the cortex, striatum, thalamus, and hippocampus of TBI compared to sham-injured animals at different time points (24 h–5 weeks). The data are given as fold changes relative to the control as mean ± SEM (sham groups, *n* = 4–8; TBI groups, *n* = 5–8). * *p* < 0.05; ** *p* < 0.01; *** *p* < 0.001 show differences considered statistically significant between the sham and TBI groups, + *p* < 0.05; ++ *p* < 0.01; +++ *p* < 0.001 show differences considered statistically significant comparing to the 24 h TBI group; # *p* < 0.05; ## *p* < 0.01; ### *p* < 0.001 show differences considered statistically significant comparing to the 4th day TBI group; & *p* < 0.05; && *p* < 0.01; &&& *p* < 0.001 show differences considered statistically significant when comparing to the 7th day TBI group; ^^^ *p* < 0.001 shows differences considered statistically significant comparing to the 2 weeks TBI group as estimated by two-way ANOVA followed by Bonferroni’s post hoc test.

*GFAP* (a marker of astroglia) expression was elevated in all studied brain structures, and the strongest changes were observed in the cortex, striatum, and hippocampus 4–7 days after injury. In the cortex, the *GFAP* upregulation began 24 h after injury, reached the highest value on day 4 and then significantly declined until the 5th week following injury (Figure 2A). For the striatum and hippocampus, *GFAP* upregulation also started 24 h after TBI and reached a maximum on the same day. For the mRNA expression of *GFAP*, two-way ANOVA confirmed a significant interaction between the considered time points and TBI in the cortex (F_4,56_ = 15.34; *p* < 0.0001) (Figure 2A), striatum (F_4,54_ = 21.30; *p* < 0.0001) (Figure 2B), and hippocampus (F_4,51_ = 3.88; *p* = 0.0080) (Figure 2D). Moreover, we observed *GFAP* upregulation in the thalamus (Figure 2C) from the 4th day and 2nd week after TBI when comparing with that in sham animals, according to Bonferroni’s post hoc analysis.

We observed a significant increase in *IBA-1* (a marker of microglia/macrophages) mRNA levels in all analyzed brain structures (cortex, striatum, thalamus, and hippocampus). The upregulation was observed in all analyzed brain areas on the 4th day after injury, except the thalamus where the mRNA increase was observed 24 h after TBI. The peak was observed on days 4–7 in the analyzed brain areas, and the level remained elevated in all cases at least to 7 days after brain injury compared to the level in sham-operated animals (Figure 2E–H). In the thalamus, the upregulation was maintained until 5 weeks after injury (Figure 2G). For mRNA expression of *IBA-1*, two-way ANOVA confirmed a significant interaction between the considered time points and TBI for the cortex (F_4,56_ = 13.64; *p* < 0.0001) (Figure 2E), striatum (F_4,56_ = 14.77; *p* < 0.0001) (Figure 2F), thalamus (F_4,56_ = 2.90; *p* = 0.0299) (Figure 2G), and hippocampus (F_4,54_ = 5.97; *p* = 0.0005) (Figure 2H).

Elevated mRNA expression of *TMEM119* (a marker of microglia) was observed in the cortex, striatum, and hippocampus (Figure 2I,J,L). In the cortex and striatum, the upregulation was first observed and it reached the highest level on the 4th day after injury (Figure 2I,J). In the hippocampus, mRNA upregulation also began 4 days after brain injury but reached a peak 4–7 days after injury, after which it decreased (Figure 2L). For mRNA expression of *TMEM119*, two-way ANOVA confirmed a significant interaction between the considered time points and TBI in the cortex (F_4,56_ = 20.89; *p* < 0.0001) (Figure 2I), striatum (F_4,55_ = 6.84; *p* = 0.0002) (Figure 2J), and hippocampus (F_4,53_ = 4.51; *p* = 0.0033) (Figure 2L).

### 2.3. Study of GFAP and IBA-1 Protein Levels in the Cortex, Striatum, Thalamus, and Hippocampus of TBI or Sham-Injured Mice at Selected Time Points

**Figure 3:**
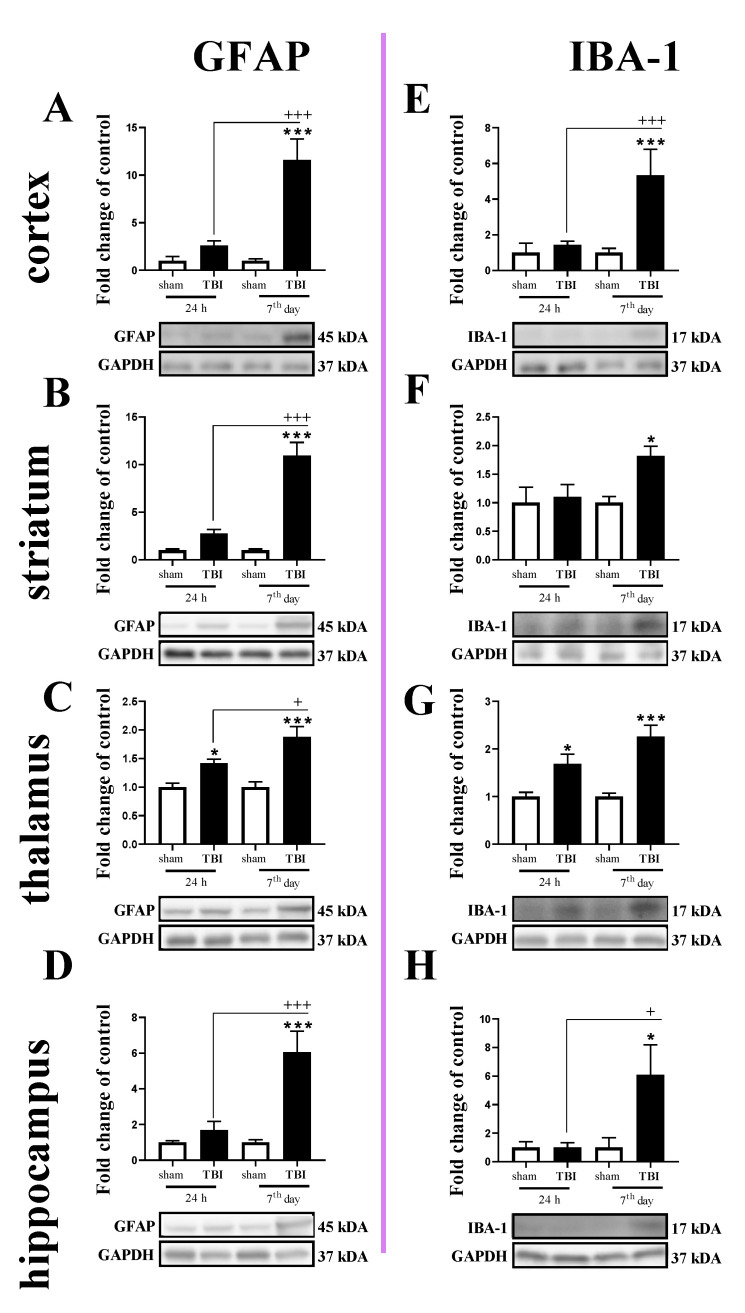
(**A**–**H**). The protein expression changes of IBA-1 and GFAP in the cortex, striatum, thalamus, and hippocampus of TBI compared to sham-injured animals at different time points (24 h and 7th days). The data are given as fold changes relative to the control as mean ± SEM (sham groups, *n* = 4–6; TBI groups, *n* = 4–6). * *p* < 0.05; *** *p* < 0.001 show differences considered statistically significant between the sham and TBI groups, + *p* < 0.05; +++ *p* < 0.001 show differences considered statistically significant comparing to the 24 h TBI group as estimated by two-way ANOVA followed by Bonferroni’s post hoc test.

The GFAP protein level was increased in all tested brain regions 7 days after TBI (Figure 3A–D). For GFAP protein expression, two-way ANOVA confirmed a significant interaction between the considered time points and TBI in the cortex (F_1,16_ = 15.13; *p* = 0.0013) (Figure 3A), striatum (F_1,19_ = 29.64; *p* < 0.0001) (Figure 3B), and hippocampus (F_1,20_ = 11.65; *p* = 0.0028) (Figure 3D). Moreover, we observed elevated levels of GFAP in the thalamus (Figure 3C) 24 h and 7 days after TBI when comparing to the levels in sham animals, according to Bonferroni’s post hoc analysis. The upregulation of GFAP protein was significantly higher on the 7th day after injury when compared to its level 24 h after injury in all subjected brain areas, as evaluated by Bonferroni’s post hoc test.

Similar to the GFAP, IBA-1 protein levels were elevated in all tested structures 7 days after TBI (Figure 3E–H). Moreover, upregulation of IBA-1 was observed in the thalamus 24 h after injury (Figure 3G). For IBA-1 protein expression, two-way ANOVA confirmed a significant interaction between the considered time points and TBI in the cortex (F_1,14_ = 10.30; *p* = 0.0063) (Figure 3E) and hippocampus (F_1,19_ = 4.56; *p* = 0.0459) (Figure 3H). Moreover, we observed elevated levels of IBA-1 in the striatum on the 7th day (Figure 3F) and thalamus (Figure 3G) 24 h and 7 days after injury when comparing with the levels in sham animals, according to Bonferroni’s post hoc analysis. 

### 2.4. Time-Dependent Study of C1q, C1s, and C1r mRNA Expression in the Cortex, Striatum, Thalamus, and Hippocampus After TBI in Mice

**Figure 4:**
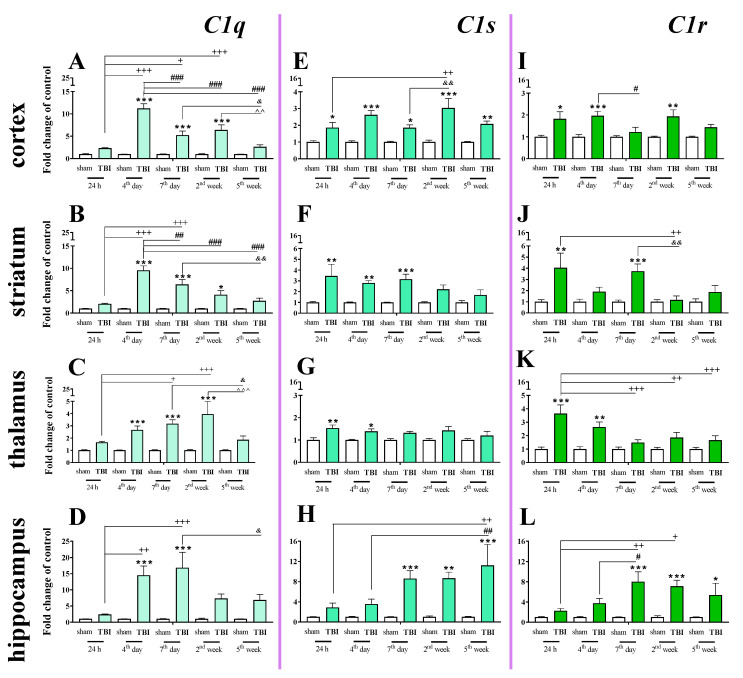
(**A**–**L**). The mRNA expression changes of *C1q, C1s*, and *C1r* in the cortex, striatum, thalamus, and hippocampus of TBI compared to sham-injured animals at different time points (24 h–5 weeks). The data are given as fold changes relative to the control as mean ± SEM (sham groups, *n* = 3–8; TBI groups, *n* = 4–8). * *p* < 0.05; ** *p* < 0.01; *** *p* < 0.001 show differences considered statistically significant between the sham and TBI groups, + *p* < 0.05; ++ *p* < 0.01; +++ *p* < 0.001 show differences considered statistically significant comparing to the 24 h TBI group; # *p* < 0.05; ## *p* < 0.01; ### *p* < 0.001 show differences considered statistically significant comparing to the 4th day TBI group; & *p* < 0.05; && *p* < 0.01 show differences considered statistically significant when comparing to the 7th day TBI group; ^^ *p* < 0.01; ^^^ *p* < 0.001 show differences considered statistically significant comparing to the 2 weeks TBI group as estimated by two-way ANOVA followed by Bonferroni’s post hoc test.

We observed a significant *C1q* mRNA increase in the cortex, striatum, and thalamus from 4 days to 2 weeks following injury and in the hippocampus from 4 to 7 days following injury. The highest level was observed on the 4th day in the cortex and striatum, on week 2nd in the thalamus and on days 4–7 in the hippocampus when comparing to sham-operated animals (Figure 4A–D). For *C1q* mRNA expression, two-way ANOVA confirmed a significant interaction between the considered time points and TBI in the cortex (F_4,55_ = 19.57; *p* < 0.0001) (Figure 4A), striatum (F_4,56_ = 11.81; *p* < 0.0001) (Figure 4B), thalamus (F_4,55_ = 3.74; *p* = 0.0092) (Figure 4C), and hippocampus (F_4,56_ = 3.67; *p* = 0.0101) (Figure 4D).

Elevated *C1s* mRNA expression was observed in the cortex, striatum, thalamus and hippocampus. In the cortex, the upregulation began 24 h after TBI and remained elevated until 5 weeks (Figure 4E). In the hippocampus, the upregulation began 7 days after TBI and also remained elevated until 5 weeks. (Figure 4H). For *C1s* mRNA expression, two-way ANOVA confirmed a significant interaction between the considered time points and TBI in the cortex (F_4,55_ = 2.69; *p* < 0.0406) (Figure 4E) and hippocampus (F_4,55_ = 3.16; *p* = 0.0206) (Figure 4H). Moreover, according to Bonferroni’s post hoc analysis, we observed elevated levels of *C1s* in the striatum (Figure 4F) and the thalamus (Figure 4G) after TBI compared with levels in sham animals. Increased *C1s* levels were observed between 24 h and 7 days after injury in the striatum and between 24 h and 4 days after injury in the thalamus.

Elevated expression of *C1r* was observed in the striatum and thalamus 24 h after brain injury. In the hippocampus, *C1r* upregulation began 7 days after TBI and remained elevated until 5 weeks (Figure 4L). The highest levels of C1r upregulation were observed 24 h after injury in the striatum and thalamus and 7 days after injury in the hippocampus (Figure 4J–L). For *C1r* mRNA expression, two-way ANOVA confirmed a significant interaction between the considered time points and TBI in the striatum (F_4,54_ = 2.94; *p* = 0.0285) (Figure 4J), thalamus (F_4,49_ = 3.07; *p* < 0.0246) (Figure 4K), and hippocampus (F_4,48_ = 3.18; *p* < 0.0214). Moreover, according to Bonferroni’s post hoc analysis, we observed elevated levels of *C1r* in the cortex (Figure 4I) 24 h, 4 days, and 2 weeks after TBI when compared with levels in sham animals.

### 2.5. C1q Localization in the Contused Tissue

**Figure 5:**
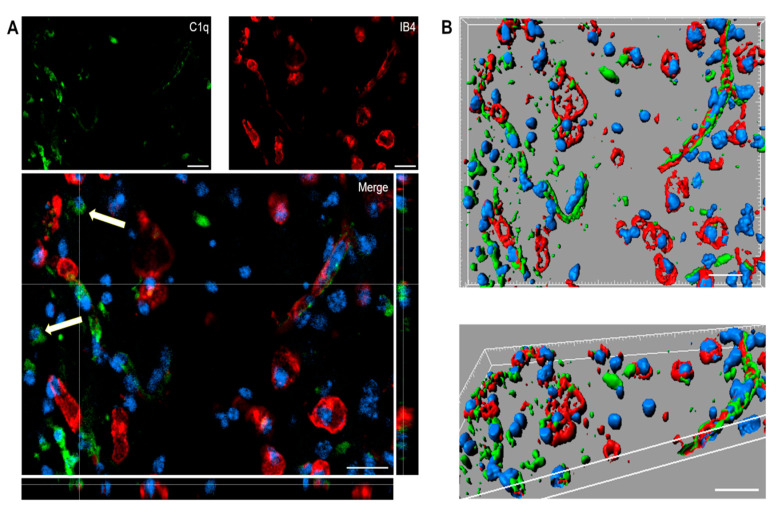
(**A**,**B**). Confocal analysis of C1q localization in the contused cortex 96 h after TBI. (**A**) C1q (green) is present on brain vessels (IB4, red) and in cell-like structures (arrows). Nuclei are in blue. (**B**) Three-dimensional image rendering shows C1q in vessels. Images are representative of at least two independent experiments. Scale bar = 20 µm.

Immunofluorescence analysis confirmed the presence of C1q in the contused cortex 96 h after TBI. Interestingly, C1q localizes to brain vessels and cell-like structures (Figure 5A). 

**Figure 6:**
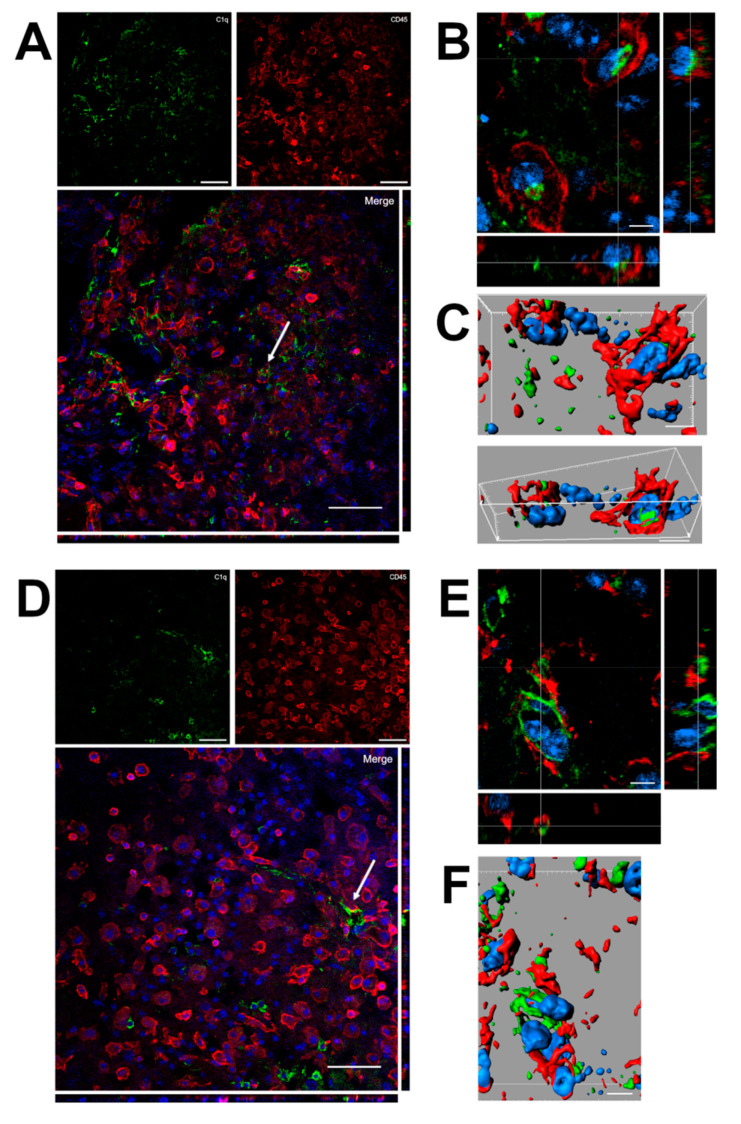
(**A**–**F**). Confocal analysis of C1q and microglia/macrophage reciprocal localization in the contused cortex 96 h after TBI. (**A**,**D**) C1q (green) does not generally colocalize with microglia/macrophages (Cd45, red) although it appears in close proximity to them (arrow in A). C1q appears with vessels-like morphology (arrow in D), as shown in Figure 5. Scale bar = 50 µm. (**B**,**C**). High magnification, three-dimensional image rendering shows C1q localization inside Cd45 positive cells. (**E**,**F**) High magnification, three-dimensional image rendering shows C1q surrounded by microglia/macrophages. Images are representative of at least two independent experiments. Nuclei are in blue. Scale bar = 5 µm.

Confocal analysis was performed of C1q and microglia/macrophage localization in the contused cortex 96 h after TBI (Figure 6). Panels A and D show that C1q does not generally colocalize with microglia/macrophages, although it localizes in close proximity to them. Instead, C1q appears with vessel-like morphology, as shown in Figure 5. Panels B and C show the high magnification, three-dimensional image rendering of C1q inside Cd45 positive cells. Panels E and F show the high magnification, three-dimensional image rendering of C1q surrounded by microglia/macrophages. Images are representative of at least two independent experiments.

**Figure 7:**
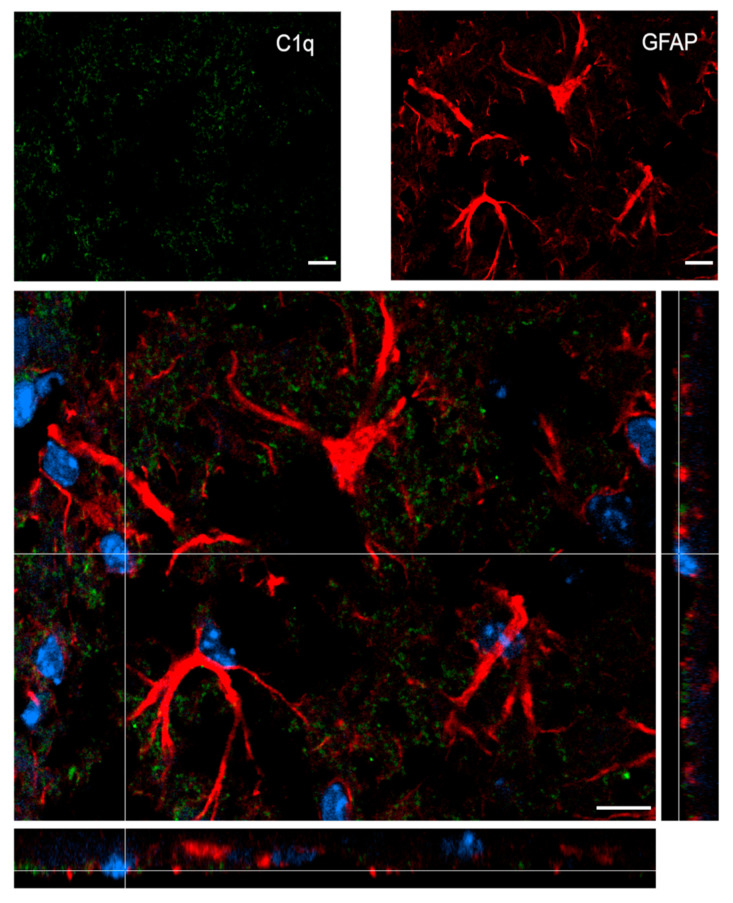
Confocal analysis of C1q and astrocyte reciprocal localization in the contused cortex 96 h after TBI. A high magnification image shows that C1q (green) does not specifically colocalize with astrocytes (GFAP, red). Nuclei are in blue. Images are representative of at least two independent experiments. Scale bar = 10 µm.

Figure 7 shows confocal analysis of C1q and astrocyte localization in the contused cortex 96 h after TBI. C1q is present along the border of the lesion, but it does not specifically co-localize with astrocytes. 

**Figure 8:**
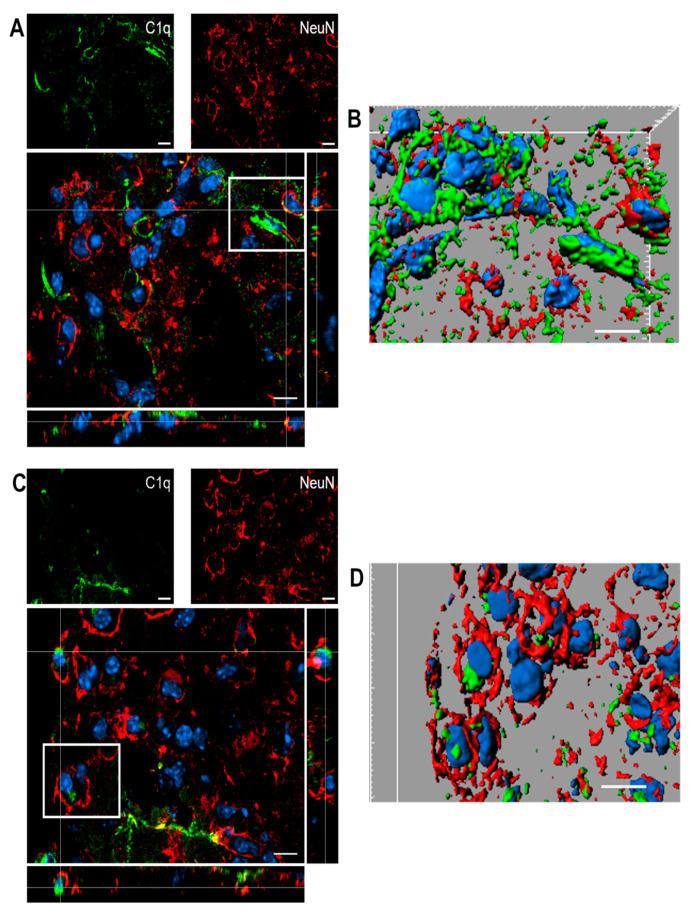
(**A**–**D**). Confocal analysis of C1q and neuron reciprocal localization in the contused cortex 96 h after TBI. (**A**,**C**) C1q (green) is present along the border of the lesion close to neurons (NeuN, red). C1q frequently localizes in close proximity to neurons. (**B**–**D**) Three-dimensional image rendering shows C1q colocalization with neurons, possibly driving apoptosis. Images are representative of at least two independent experiments. Nuclei are in blue. Scale bar = 10 µm.

Figure 8 shows confocal analysis of C1q and neuronal localization in the contused cortex 96 h after TBI.

### 2.6. Time-Dependent Study of Mannose Binding Lectin A and C, Collectin 11, Ficolin A and Ficolin B mRNA Expression in the Cortex, Striatum, Thalamus, and Hippocampus After TBI in Mice

**Figure 9:**
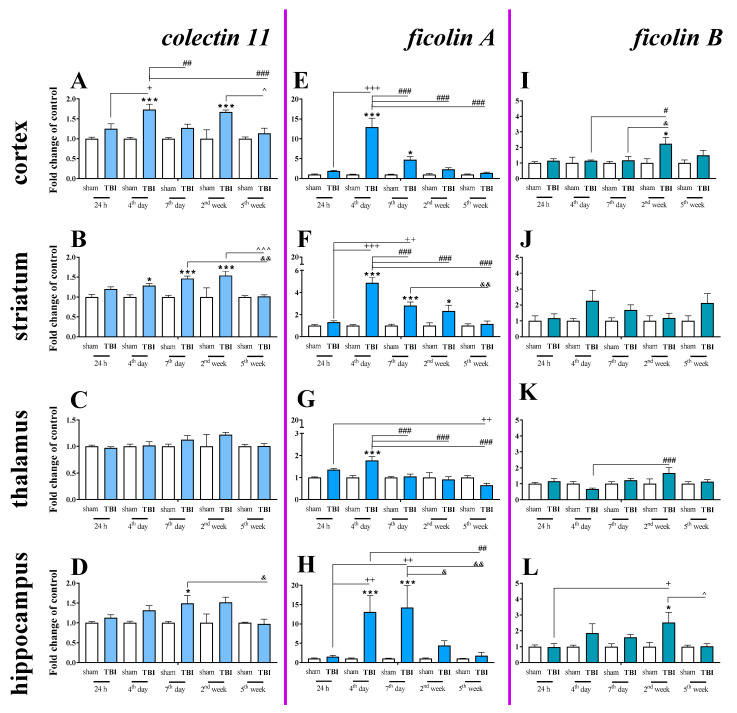
(**A**–**L**). The mRNA expression changes of *collectin 11, ficolin A*, and *ficolin B* in the cortex, striatum, thalamus, and hippocampus of TBI compared to sham-injured animals at different time points (24 h–5 weeks). The data are given as fold changes relative to the control as mean ± SEM (sham groups, *n* = 3–8; TBI groups, *n* = 4–8). * *p* < 0.05; *** *p* < 0.001 show differences considered statistically significant between the sham and TBI groups, + *p* < 0.05; ++ *p* < 0.01; +++ *p* < 0.001 show differences considered statistically significant comparing to the 24 h TBI group; # *p* < 0.05; ## *p* < 0.01; ### *p* < 0.001 show differences considered statistically significant comparing to the 4th day TBI group; & *p* < 0.05; && *p* < 0.01; show differences considered statistically significant when comparing to the 7th day TBI group; ^ *p* < 0.05; ^^^ *p* < 0.001 show differences considered statistically significant comparing to the 2 weeks TBI group as estimated by two-way ANOVA followed by Bonferroni’s post hoc test.

Basal mRNA levels of *collectin 11* (Figure 9A–D), *ficolin A* (Figure 9E–H) and *ficolin B* (Figure 9I–L) were detected in the brains of control mice, but *mannose binding lectin A* and *mannose binding lectin C* mRNA were not detected (Table 1). 

Moreover, changes in *collectin 11* (Figure 9A–D), *ficolin A* (Figure 9E–H) and *ficolin B* (Figure 9I–L) mRNAs were also detected after brain injury. Following TBI, elevated *collectin 11* mRNA levels were observed in the cortex and striatum, beginning on 4 days after TBI and were observed until 2 weeks following injury when compared to the levels observed in the sham group (Figure 9A,B). We did not observe any changes in expression of *collectin 11* in the thalamus (Figure 9C). For *collectin 11* mRNA expression, two-way ANOVA confirmed a significant interaction between the considered time points and TBI in the cortex (F_4,56_ = 3.54; *p* = 0.0121) (Figure 9A) and striatum (F_4,56_ = 3.24; *p* = 0.0184) (Figure 9B). Moreover, according to Bonferroni’s post hoc analysis, we observed elevated *collectin 11* levels in the hippocampus (Figure 9D) on the 7th day after TBI when compared to the levels in sham animals.

After TBI, *ficolin A* mRNA levels were significantly increased in all analyzed brain structures (the cortex, striatum, thalamus, and hippocampus) in comparison to the levels in sham-operated animals. The upregulation was observed in all brain structures on the 4th day after TBI and also peaked on the 4th day after injury in the cortex, striatum, and thalamus (Figure 9E–G). However, the highest hippocampal *ficolin A* mRNA levels were observed on days 4–7 after injury (Figure 9H). For *ficolin A* mRNA expression, two-way ANOVA confirmed a significant interaction between the considered time points and TBI in the cortex (F_4,56_ = 14.38; *p* < 0.0001) (Figure 9E), striatum (F_4,56_ = 13.41; *p* < 0.0001) (Figure 9F), thalamus (F_4,56_ = 7.28; *p* < 0.0001) (Figure 9G), and hippocampus (F_4,52_ = 4.04; *p* = 0.0063) (Figure 9H). 

Moreover, according to Bonferroni’s post hoc analysis, we observed elevated levels of *ficolin B* in the cortex and hippocampus after TBI (Figure 9I,K,L) when comparing to levels in sham animals. 

### 2.7. Study of C1q, C1s, C1r, Mannose Binding Lectin A, Mannose Binding Lectin C, Collectin 11, Ficolin A, and Ficolin B mRNA Expression in Primary Microglial Cultures After LPS Treatment

**Figure 10:**
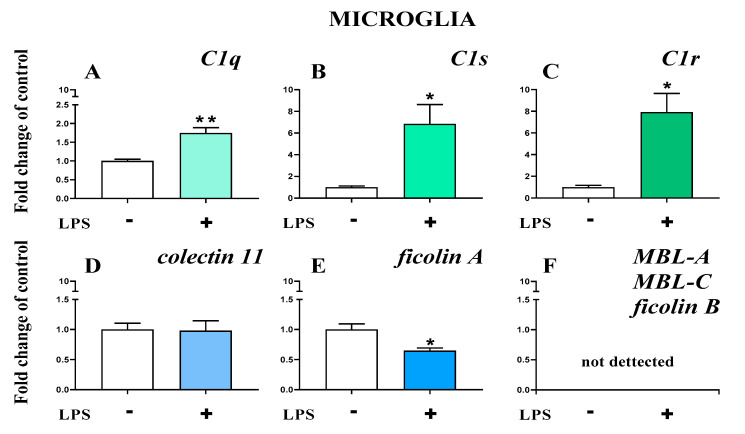
(**A**–**F**). The changes in initiators of classical pathway [green]: *C1q*, *C1s*, *C1r*; lectin pathway [blue]: *mannose binding lectin A* and *C* (*MBL-A*, *MBL-C*), *collectin 11*, *ficolin A*, and *ficolin B* mRNA expression in primary microglial cultures. The data are the mean ± SEM (*n* = 3). * *p* < 0.05; ** *p* < 0.01 indicate significant differences between non-stimulated (-) and LPS-stimulated (+) cultures.

The basal mRNA levels of *C1q* (Figure 10A), C1s (Figure 10B), and C1r (Figure 10C) were assessed in primary microglia cell cultures. After LPS stimulation, a significant increase in all the initiators of the classical pathway was observed (Figure 10A–C). In primary microglial cell cultures, after LPS stimulation, increased mRNA levels were observed for C1q (1.74 ± 0.14 (t4 = 4.96; *p* = 0.0077)) (Figure 10A), C1s (6.84 ± 1.79 (t4 = 3.25; *p* = 0.0314)) (Figure 10B), and *C1r* (7.92± 1.72 (t_4_ = 4.01; *p* = 0.0160)) (Figure 10C). 

Further, the basal mRNA levels of *collectin 11* (Figure 10D) and *ficolin A* (Figure 10E) but not *ficolin B*, *mannose binding lectin A*, or *mannose binding lectin C* (Figure 10F) were assessed in primary microglial cell cultures. After LPS stimulation, a significant decrease in *ficolin A* (0.65 ± 0.44 (t_4_ = 3.4; *p* = 0.0273)) (Figure 10E) was observed.

## 3. Discussion

The contribution of the complement system to acute brain injury is well known. While it is widely recognized that circulating complement cascade components enter the brain through a damaged blood-brain barrier, the possibility that the brain itself may respond to injury with a local synthesis of complement components has been scarcely explored. Herein, we have documented the ipsilateral spatiotemporal activation and mRNA expression of initiators of the classical (*C1q*, *C1s*, *C1r*) and lectin (*collectin-11*, *ficolin A*, *ficolin B*) complement pathways in murine brain structures after TBI. Importantly, our study has revealed, for the first time, that these complement changes are detected not only in the cortex but also in other brain regions, such as the striatum, hippocampus, and thalamus. In parallel, we have detected significant changes in the mRNA levels of a wide spectrum of cellular markers for neutrophils, Cd8+ T cells, astroglia, and microglia/macrophages. Our data added to the knowledge that C1q, C1s and C1r (the C1q complex), and ficolin A are important factors in the brain, and we assert that the roles of these proteins should be fully elucidated. 

Interestingly, C1q and its complex components are detected in strongly activated microglial cells. Moreover, our in vitro experiments using primary cells cultures confirmed that microglial cells can synthesize classical pathway initiators but are unlikely to be responsible for elevated levels of lectin pathway initiators. Therefore, strategies that modulate microglial activation and/or blockade of the C1q complex may represent a promising opportunity for effective therapies in brain damage. It has been previously shown that C1-inhibitor protects from focal brain trauma in mice by reducing thrombo inflammation and diminishing upregulation of proinflammatory factors, such as CCL2, CCL3, IL-beta, and tumor Necrosis Factor alpha (TNF-α) [15]. Previous studies also show that administration of C1-inhibitor shortly after TBI, attenuates cognitive deficits and histological damage [16]. Moreover, it was reported that C1-inhibitor attenuates neurobehavioral deficits and reduces contusion volume after TBI in mice [17]. Therefore, the role of the C1q complex requires in-depth pharmacological investigation.

Brain injury is known to evoke an inflammatory reaction that involves several interrelated mechanisms, including release of intracellular components from damaged cells to the parenchyma, production of complement factors and cytokines, activation of brain-resident cells, and recruitment of peripheral immune cells into the brain [18,19]. Many of these processes influence each other, leading to complex interactions. Over the last years, because of heterogeneous changes observed in patients with TBI, different animal models have been developed with the aim of reproducing a complex clinical condition and to obtain tools to better recognize the underlying pathophysiology [20]. Among them, 3 models are widely used in particular: CCI [13,21,22], fluid percussion injury (FPI) [23] and impact acceleration model of diffuse traumatic brain injury (DTBI) [24]. CCI is a dynamic direct impact injury which replicates clinical brain injury with skull deformation and related cortical compression. It offers a very good control of injury parameters such as velocity of impact, depth, and angle, resulting in a highly reproducible impact [13,21,22], a potential advantage over other models such as FPI. A few changes measured in our study using CCI are similar to those observed by others in FPI model, for example after one week both injuries show enhanced cortical and/or hippocampal infiltration and/or activation of GFAP+, IBA-1+, and TMEM+ cells [7,25,26,27]. Importantly, CCI reproduces changes reported in clinical head injuries and documented in several studies such as: concussion, contusion, hemorrhage, traumatic axonal injury and cortical inflammatory changes, brain edema, elevated intracerebral pressure, reduced cortical perfusion, decreased cerebral blood flow, neuroendocrine, and both inflammatory and metabolic changes [10,20,28,29]. Therefore, learning about neuroimmune interactions from animal models of TBI may foster the development of future therapies. Knowledge about the brain regions affected by the cascade of events triggered by TBI is limited; therefore, we decided to expand our studies to other brain areas. The results demonstrated that cellular changes in CCI occurred not only in the cortex (which was the injury site corresponding to the most expected changes) but also in the striatum, hippocampus, and thalamus. We showed the spatiotemporal mRNA expression of cellular markers and complement initiators in different brain areas (cortex, striatum, thalamus, and hippocampus), which were previously suggested to be especially susceptible in TBI [30,31,32]. It was previously demonstrated in a closed head diffuse injury rat model that TBI leads to wide, long-lasting microstructural alterations in white and gray matter [33]. Moreover, the same brain areas we studied (cortex, striatum, thalamus, and hippocampus) appeared to be especially susceptible to ongoing post-TBI pathology in previous studies, which was confirmed by volumetric changes in these areas and/or significantly higher activation of microglia/macrophages when compared to the sham group (up to 30 days) [33]. Additionally, after TBI, positron emission tomography confirmed an increased level of inflammation in the thalamus [10].

One of the most important first-line players at the sites of injury are neutrophils [34]. Our results showed that the mRNA level of *Cd177* (a marker of neutrophils) is upregulated and peaks on the 4th day after brain damage in the cortex and also in the striatum and hippocampus to a lesser extent. This observation is in agreement with previous studies, which show that recruitment of neutrophils into the injured cortex occurs over a period of approximately 3 days [6]. In early stages of injury, activated neutrophils bind to the endothelium and platelets and as a consequence, hinder the blood flow through vessels [35]. Moreover, as a source of several inflammatory mediators, such as proinflammatory cytokines, reactive oxygen species (ROS) and matrix metalloproteinases (MMPs), neutrophils contribute to the development of severe complications after TBI [36,37]. Conversely, some studies have shown that neutrophils also affect nerve repair by releasing transforming growth factor β (TGF-β), nerve growth factor (NGF), IL-4, and IL-10 [6], and that is why the role of those cells is rather dualistic. Neutrophils are regarded as important short-lived players in brain injury, in contrast to Cd8+T cells [38,39,40]. After TBI in mice, an increase in Cd8+ T cells can be observed in the cortex up to 8 weeks after injury [41]. Additionally, it has been shown, in experimental stroke, that mice display improved behavioral function and decreased neuronal loss following the depletion of Cd8+T cells [42]. Our data confirm the presence of Cd8+ T cells in the cortex both during early and later phases; however, the maximal activation levels are reached at later time points. Importantly, our results give evidence that the observed changes are not limited to the cortex but are also present in the striatum, thalamus, and hippocampus. Based on the literature [41], one may believe that after TBI, Cd8+ T cells promote secondary neuronal and myelin degeneration not only in the cortex but also in other brain regions. Oligodendrocytes are required for producing and maintaining myelin throughout the brain. Our data show that after TBI, *Olig2+*, which is a marker of immature oligodendrocytes, is only briefly and slightly upregulated in the cortex, striatum, and hippocampus after injury, which is in agreement with others [43]. After TBI, decreased activity of CC-1+, which is a marker of mature oligodendrocytes, has been reported in the literature [43], and this is in line with our results showing that new immature oligodendrocytes do not form after TBI, which contributes to the gradual degeneration of myelin. In contrast, TBI has been shown to evoke the activation of astrocytes, the most abundant cell type in the brain [44,45]. This is in agreement with our results showing the increased expression of *GFAP*, the astroglial activation marker, in the cortex, striatum, thalamus, and hippocampus. Therefore, the serum GFAP level appears to be a good prognostic marker of TBI [46]. The literature shows that astrocytes can have some protective functions in the injured brain, e.g., through the release of neurotrophic factors [47,48]. Homeostatic astrocytes are critical for maintaining normal function of the blood-brain barrier [49]; however, the major result of astrocyte activation is the formation of glial scars, which function as a barrier to axonal regeneration and extension [47,50]. Herein, we observed significant *GFAP* level changes in the brain starting early after injury and lasting up to 5 weeks. In a very recent study, it was shown that monitoring IBA-1 in serum might provide clinically relevant insights into the underlying TBI pathophysiology [51]. The IBA-1 is a monocytes/microglia/macrophages-specific calcium-binding protein that participates in membrane ruffling and phagocytosis of those cells. Among IBA-1 positive cells, activated monocytes infiltrate into the brain after injury and propagate breakdown of the BBB by secreting a variety of inflammatory mediators. Recently Ly6C^lo^ non-classical monocytes were suggested to be necessary for the recruitment of neutrophils [52], while CD115^+^Ly6C^−hi^CD62^+^CCR2^hi^ classical monocytes, infiltrate into the injured brain, and become tissue macrophages [53]. Macrophages and microglia are considered to be important players in maintaining brain homeostasis in the response to injury [54,55]. Long-term increased IBA-1 levels after brain injury indicate that those cells become activated and/or infiltrate to injured regions, both in early and late phases [56]. These processes may lead to neuroinflammation, which may have some protective functions but also may cause secondary brain injury and neurodegeneration [56,57]. Our current study indicates that the upregulated mRNA and protein levels of IBA-1 reflect long-term activation in the cortex and other brain regions, such as the striatum, hippocampus, and thalamus. The role of microglia in TBI may also be confirmed by the presence of transmembrane protein 119 (TMEM119), which is exclusively expressed on those cells [58,59,60]. Our data has revealed for the first time that following TBI, *TMEM119* is upregulated for up to 2 weeks in the cortex, what may be connected with loss of microglia homeostatic functions. Therefore, future studies are required to determine the exact role of microglia during TBI, including the role in complement activation. It has already been shown that C1q can be produced by microglia cells [61,62]; however, our data provide evidence that microglia are also a source of C1s and C1r. In sum, our results suggest that microglia possess the machinery for production of the C1q complex, which can be triggered after their activation. Whether or not this is related to activation of the complement cascade or if C1q possess independent actions is still to be clarified. This may be similar to what was recently shown for MBL, which can exert a direct deleterious effect on ischemic brain endothelial cells independently from complement activation [63].

C1q is a recognition and initiator molecule of the classical complement activation pathway, which binds to targets (immune complexes), leading to serine protease C1r activation, which cleaves and activated C1s—its substrate. C1q is synthesized by brain macrophages and microglia [12,64,65] and is involved in the initiation of those cells activation in the extrinsic plasma, during the occurrence of central nervous system (CNS) diseases characterized by BBB impairment [12]. Moreover, C1q is synthesized by activated microglia/macrophages and because of that, it is considered to be the marker of their activation [66], which is in agreement with the results of our IHC and in vitro analysis. A previous in vitro study has shown that C1q evokes calcium signaling in microglia/macrophages and triggers the release of cytokines [12]. Therefore, it appears that this defense protein complex is likely to contribute, in an autocrine/paracrine way, to microglia/macrophages homeostasis. Under physiological conditions, the level of C1q is low in the CNS, but it increases significantly after damage or during infections, which is evident in animal model studies of blood-brain barrier dysfunction [64], transient global cerebral ischemia [65], virus infections [67], and spinal cord injury [68]. Importantly, C1q upregulation has been reported in humans during neurodegenerative impairments, such as Alzheimer’s disease, frontal temporal dementia, West Nile Virus infection, and schizophrenia [69,70,71,72]. Additionally, studies in C1q-deficient mice reveal that C1q has a detrimental effect on the development of CNS diseases, such as Alzheimer’s [73] and prion [74] diseases. Further, it has been shown that C1q deficiency improves histological and functional locomotor outcomes after spinal cord injury [75]; however, motor function recovery has not been observed after brain injury [76]. It has been shown that accumulation of C1q on synapses within the hippocampus occur in parallel with synapse loss 30 days post TBI [77]. It was also demonstrated that C1q-dependent complement activation contributes to hypoxic-ischemic brain injury in mice, and deletion of the *C1q* gene confers significant and long-lasting neuroprotection [78]. However, C1q is not only the classical complement pathway initiator, but it has also been implicated in several functions that may be independent of the complement cascade, including the regulation of synaptic pruning, protection against neurotoxicity, and promotion of angiogenesis [79]. In the TBI model, we also observed a significant upregulation in *C1q* mRNA with major changes on days 4–7 and 2 weeks in the thalamus, and this enhancement was maintained up to 2 weeks after injury in all studied brain regions (except the hippocampus). Importantly, these changes occurred in parallel with the *IBA-1* mRNA upregulation, suggesting that microglia/macrophages are the source of C1q. This is confirmed by IHC results, showing the co-localization of C1q and Cd45. In 2017, Thielens et al. proposed that the role of C1q in the brain may not be related only to activation of the classical pathway [80]. There are different ways by which C1q can bind to the receptors and target the cells: by the collagen-like tail (cC1qR) and the globular head of the molecule (gC1qR), also by different ligands like heparan sulfate [80,81]. Therefore, C1q has also been reported to induce expression of adhesion molecules [82], enhance the release of proinflammatory cytokines [83], recognize apoptotic endothelial cells [84], and stimulate expression of genes in neurons that are associated with neuroprotection [85]. Interestingly, an in vitro study showed that C1q suppresses the LPS-evoked production of proinflammatory factors TNF-α, IL-1β, and IL-6, suggesting its homoeostatic and anti-inflammatory role in the CNS [86]. Importantly, in vitro studies show that C1q, in the absence of C1r and C1s, induces gene expression that is critical for neuronal survival and neurite outgrowth [85,87] and protects against oligomeric and fibrillar Aβ-induced (amyloid beta induced) neuronal death [79]. However, after TBI, the mRNA levels of *C1q*, *C1r*, and *C1s* increase, which may be one of the reasons for secondary injury development after TBI. Moreover, these changes are not only observed in the cortex but are also observed in other brain regions, such as the striatum, hippocampus, and thalamus, and may contribute to the progression of neurodegeneration. Our results are in line with those obtained in FPI model in injured cortex and hippocampus [88]. In addition, our study provides the first evidence that strong changes appear in striatum, a structure involved in movement planning, as well as in cognition and reward processes and in the thalamus, an area important for consciousness, arousal, cognition, behavior, working memory, executive function, motor control, sustained, and vigilant attention [89]. Noticeable changes are also detected in the hippocampus, the brain region responsible for cognition, memory deficits, and epileptic seizures associated with TBI [31]. Thus, our data demonstrate that in these important areas of the brain, there is a strong activation of the initiators of the classical complement activation pathway, paving the way to the development of novel effective pharmaceutical agents. It appears that C1q is neuroprotective only when acting independently (without C1r and C1s); however, during TBI, the entire C1q complex appears to be involved. Recently, the C1-inhibitor has been proposed as an attractive candidate for treatment of neurological disorders associated with inflammation [15], including stroke [90] and TBI [17]. In our opinion, because there are many diseases wherein C1q-complex targeting may be successful, further studies addressing the downstream consequences of activation of this multifunctional complex will be critical to develop therapy for TBI and other neuroinflammation-related diseases. 

While the C1q-complex components may be synthesized in the brain, MBL is mainly produced by liver and is the typical source of complement proteins [91,92]. Our results give evidence for a lack of *MBL-A* and *MBL-C* mRNA in all studied brain structures, even after TBI. Further, our in vitro experiments confirm the lack of MBLs biosynthesis by primary microglia, even after their activation. However, it is conceivable that MBL can enter the brain from the blood through the injured blood-brain barrier and may act as a chemoattractant for immune cells [93]. Although MBL is not synthesized in the brain, it plays an important role after brain injury. Notably, MBL knockout mice or mice receiving an MBL inhibitor show attenuated motor deficits and reduced neuronal cell loss following injury [94]. Unlike MBL, collectin-11 shows cerebral synthesis and its mRNA expression is activated by TBI. It has been previously shown that expression of collectin 11 rapidly increases in the post-ischemic period and colocalizes with complement proteins deposited along the basolateral surface of the proximal renal tubule [95]. Our experimental results show, for the first time, that there is slight upregulation of *collectin-11* mRNA in the mouse cortex, hippocampus, and striatum after TBI. Moreover, since there is no upregulation of *collectin-11* mRNA in primary microglia after their activation, those cells may not be the main source of collectin-11 in the brain. Moreover, it has been recently reported that collectin-11 knock-out mice do not show an improved sensorimotor response compared to wild type mice [96], which suggests that this initiator of the lectin pathway is not crucial in TBI. 

We have previously demonstrated that ficolin-1, -2, and -3 are present in the contused human brain; however, their role remains unclear. Therefore, ficolins were investigated in the murine model herein. In humans, ficolin-1 can be detected close to vasculature and in brain parenchyma, while ficolin-2 and -3 are predominately identified at low levels in proximity to brain vessels [29]. The levels of ficolin-2 and -3 increase (2.2 and 6.0 times) in brains after TBI [29], but ficolin-1 levels remain unchanged. In mice, only two types of ficolins, called ficolin A (related to human ficolin 2) and ficolin B (related to human ficolin 1), are present [97,98]. Our results indicate that after TBI in mice, *ficolin A* mRNA is upregulated in the early phase, and slight increases in *ficolin B* mRNA are observed later. The changes in ficolin B are very low in mice, so it may be biologically irrelevant, which fits well with the previous results obtained in humans [29]. The transcriptome database [99] reports that ficolin A is expressed almost exclusively in microglia and macrophages, in contrast to ficolin B, which can be produced by neutrophils [100]. This is in line with our in vitro results showing that ficolin A but not B can be produced by microglial cells. Moreover, the transcriptome database [99] supports our in vitro results showing the lack of *ficolin B* mRNA in microglia, even after their stimulation. Importantly, recently published findings give evidence that after TBI, ficolin A knockout mice have better outcome scores when compared to wild-type mice [96], suggesting that ficolin A may play a role in TBI.

Based on our results and available literature, we conclude that role of complement in TBI is greater than previously assumed, with long-term changes observed not only in the cortex, but also in the striatum, hippocampus, and thalamus. Especially interesting is a fact that not only C1q, but also C1s and C1r can be produced by activated microglial cells under TBI. Therefore, strategies that modulate microglia/macrophages activation and/or blockade of the C1q complex might represent a promising opportunity for novel, effective therapies in brain damage. As mentioned above, C1-inhibitor has been shown to act as an important tool in reducing functional and neuropathological consequences of TBI [15,17]. Importantly, this molecule is already available for clinical use in patients suffering from hereditary angioedema [101]. In addition, other novel inhibitors are presently being designed to block C1q such as a functional C1q antibody that binds to the globular head domain of C1 and prevents activation of C1r/s. Preliminary research indicates that this antibody enters the brain and decreases synapse loss and cognitive dysfunction [72]. In light of our results, it seems that using such a functional C1q antibody in TBI models might contribute to a deeper understanding of pathomechanism and will bring us closer to developing more effective therapy. Currently developed C1r/s inhibitors could also hold promise in future research if brain penetrant compounds would be established. Moreover, our research highlights significant changes in ficolin A not only in the cortex but even in other brain areas following TBI. So far, ficolin A and ficolin 1 have been considered as plasma markers of severity after brain injury, including in clinic [102]. Ficolin 1 is considered as a target for autoimmune diseases therapy [103]. However, further more research is still required to prove the anti-inflammatory effect of anti-ficolin 1 monoclonal antibody in CNS, especially in the light of current results showing the interest of performing such studies in TBI models. Overall, a deeper understanding the role of complement factors and their spatial and temporal fluctuations will be pivotal for development of novel therapeutic strategies for TBI.

## 4. Materials and Methods 

### 4.1. Animals

Procedures involving animals and their care were conducted in conformity with institutional guidelines in compliance with national and international laws and policies (prot.9F5F5.81 authorization n° 753/2017-PR). All animals had free access to food and water and were maintained at a temperature of 22 ± 2 °C and a relative humidity of 55 ± 10% on a 12 h light/dark cycle. We used male 9 week old C57BL/6J mice weighing 22–28 g (purchased from Charles River, Calco, Italy). Pregnant C57BL/6J female mice (purchased from Charles River, Cologne, Germany) were used to obtain 1-day-old mouse pups for primary glial cell culture studies.

### 4.2. Experimental Traumatic Brain Injury (TBI Model)

Inhalation anesthesia (isoflurane—induction, 3% and maintenance, 1.5%) in N_2_O/O_2_ (70/30%) was used to anesthetize mice that were immobilized in a stereotaxic frame. Animals were exposed to a craniotomy on the left side of the skull and then to TBI through the controlled cortical impact using a previously established procedure [13,21,22]. The TBI was performed using a 3 mm diameter rigid impactor driven by a pneumatic piston rigidly mounted at an angle of 20° from the vertical plane and applied vertically to the exposed dura mater, between bregma and lambda, over the left parieto-temporal cortex. The impactor velocity of 5 m/s and depth of 1 mm were set, which resulted in severe brain injury. After CCI, craniotomy and scalp stitching were performed. Sham-injured mice were exposed to the same anesthesia and surgery without brain injury. No animals died after surgery. Typical TBI mice present a mean cortical lesion of 17 mm^3^ 6 weeks after injury [3,96].

### 4.3. Primary Microglial Cell Cultures

Primary microglial cell cultures (prepared from the cerebral cortex obtained from newborn C57BL/6J mice) were used to perform in vitro studies, as described in our previous papers [104,105]. The cells were plated in culture medium consisting of high-glucose GlutaMAX^TM^ DMEM supplemented with 10% heat-inactivated fetal bovine serum, 0.1 mg/mL streptomycin and 100 U/mL penicillin (Gibco, Gaithersburg, MD, USA) on poly-L-lysine-coated 75-cm^2^ culture flasks (at a seeding density of 3 × 10^5^ cells/cm^2^) and grown in an incubator with a humidified atmosphere (37 °C, 5% CO_2_ in air). The culture medium was replaced with fresh medium every 4 days. The microglial cells, which were loosely attached to the monolayer, were gently harvested by shaking at 70 revolutions per minute (RPM) for 1 h and then at 90 RPM for 15 min and then were centrifuged (800 RPM—10 min) on the 16th day of culture. The viability of cells in culture was determined using Trypan blue (Bio-Rad, Warsaw, Poland) exclusion method. The primary microglial cultures were treated with lipopolysaccharide (LPS, 100 ng/mL, Sigma-Aldrich, St. Louis, MO, USA) for 24 h. Cells were seeded at a density of 2 × 10^5^ cells/well on 24 well plates and cultured for 24 h followed by analysis of the mRNA levels. IBA-1 (microglial marker) was used to assess cell specificity. A minimum essential number of newborn animals were used for culture generation. Although LPS stimulation does not directly imitate TBI-induced inflammation, we decided to use it since it is one of the most widely-used pro-inflammatory factor to study immune response of glial cells. As previously reported [106], some of the genes up-regulated by LPS are also strongly expressed by microglia after TBI, as shown in ex vivo studies. Additionally comparison of LPS stimulation with that obtained using interferon-γ (IFNγ) and tumor necrosis factor-α (TNFα) showed that both treatments increased numerous inflammatory genes, however the changes observed after LPS were most pronounced [107].

### 4.4. Biochemical Analysis

#### 4.4.1. RT-qPCR

To assess gene expression, tissue obtained from selected brain ipsilateral areas, including cortex (with all the tissue above the rhinal fissure), striatum, thalamus, and hippocampus, and cell culture lysates were used. Samples from ex vivo experiments were dissected from sham and TBI mice at five time points (24 h, 4 days, 7 days, 2 weeks and 5 weeks), immersed in RNA-later (Ambion, Inc, Austin, TX, USA) and frozen at −80 °C before use. For RT-qPCR, total RNA was extracted [108] with TRIzol reagent (Invitrogen, Carlsbad, CA, USA) as previously described [109]. The total RNA concentration was measured using a DeNovix DS-11 Spectrophotometer (DeNovix Inc., Wilmington, DE, USA). Reverse transcription was performed on 1000 ng (for tissue) or 500 ng (for cultured cell) of total RNA using Omniscript Reverse Transcriptase (Qiagen Inc., Hilden, Germany) at 37 °C for 60 min. The resulting cDNA was diluted 1:10 with H_2_O. RT-qPCR was performed using Assay-On-Demand TaqMan probes according to the manufacturer’s protocol (Applied Biosystems, Foster City, CA, USA) and was run on an iCycler device (BioRad, Hercules, Warsaw, Poland). The following TaqMan primers were used: Mm00446968_m1 (hypoxanthine guanine phosphoribosyl transferase, *Hprt*), Mm00479862_g1 (allograft inflammatory factor 1, *IBA-1*), Mm00525305_m1 (transmembrane protein 119, *TMEM119*), Mm01253033_m1 (glial fibrillary acidic protein, *GFAP*), Mm00503537_m1 (*Cd177*), Mm01210556_m1 (oligodendrocyte transcription factor 2, *Olig2*), Mm01182107_g1 (*Cd8*), Mm00432142_m1 (complement component 1, q subcomponent, alpha polypeptide, *C1qa*), Mm00663210_mH (complement component 1, s subcomponent 1, *C1s1*), Mm04206253_g1 (complement component 1, r subcomponent A, *C1ra*), Mm01289834_m1 (*collectin 11*), Mm00484287_m1 (*ficolin A*), Mm01332438_m1 (*ficolin B*), Mm00495413_m1 (*mannose binding lectin A, MBL-A*), and Mm00487623_m1 (*mannose binding lectin C, MBL-C*). Expression of the *Hprt* transcript (a housekeeping gene) was quantified to control for variations in the amount of cDNA. The cycle threshold values were calculated automatically using iCycler IQ 3.0 software with the default parameters. The abundance of RNA was calculated as 2^−(threshold cycle)^.

#### 4.4.2. Western Blotting

Ipsilateral cortical, striatal, thalamic, and hippocampal tissues harvested 24 h and 7 days after TBI or sham procedure were used for our study. The tissue was placed in radioimmunoprecipitation assay buffer (RIPA) buffer supplemented with a protease inhibitor cocktail (Sigma-Aldrich, St. Louis, MO, USA). All the samples were cleared by 14,000× *g* centrifugation for 30 min at 4 °C. The total protein concentration was measured using the bicinchoninic acid method. Protein samples were heated for 8 min at 98 °C in loading buffer (4× Laemmli Buffer, Bio-Rad, Warsaw, Poland). Samples were then loaded on 4–15% Criterion TGX precast polyacrylamide gels (Bio-Rad, Warsaw, Poland) to carry out electrophoresis. Samples were then transferred to Immun-Blot polyvinylidene fluoride (PVDF) Membranes (Bio-Rad, Warsaw, Poland) by semidry transfer (30 min, 25 V). Membranes were blocked with 5% nonfat dry milk (Bio-Rad, Warsaw, Poland) in TBST (Tris-buffered saline with 0.1% Tween 20) for 1 h, washed with TBST buffer, and incubated with the following commercially available primary antibodies overnight at 4 °C: mouse anti-GAPDH (1:5000; Merck, Darmstadt, Germany), rabbit anti-IBA-1 (1:500; Novus Biologicals, Centennial, USA), and rabbit anti-GFAP (1:10,000; Novus Biologicals, Centennial, CO, USA). The membranes were then placed in horseradish peroxidase-conjugated anti-rabbit or anti-mouse secondary antibodies (Vector Laboratories, Burlingame, CA, USA) at a dilution of 1:5000 for 1 h at room temperature (RT). The primary and secondary antibodies were dissolved in SignalBoost Immunoreaction Enhancer Kit (Merck, Darmstadt, Germany) solutions. Membranes were washed in TBST. Immune complexes were detected by the Clarity Western enhanced chemiluminescence (ECL) Substrate (Bio-Rad, Warsaw, Poland) and visualized using a Fujifilm LAS-4000 Fluor Imager system. Quantification of the bands was performed using the Fujifilm Multi Gauge system. The membranes are available for review in the Supplementary Materials file (Supplementary 1).

#### 4.4.3. Immunofluorescence and Confocal Analysis

Immunofluorescence was performed on 20 µm coronal sections in the brain ipsilateral cortex. After thorough washing with 0.01 M phosphate-buffered saline (PBS), sections were incubated for 1 h at RT with blocking solution containing 10% normal goat serum (NGS) and 0.3% Triton X-100 and then with rabbit anti-mouse C1q (1.177 mg/mL, 1:500; Abcam, Cambridge, UK). Positive cells were stained with an Alexa 488-conjugated goat secondary antibody (4 µg/mL, Life Sciences, Hercules, CA, USA). Sections were then incubated for 1 h with the following primary antibodies: mouse anti-mouse NeuN (10 μg/mL, 1:250; Merck Millipore; Burlington; MA; USA), goat anti-mouse glial fibrillary acid protein (GFAP, 0.5 μg/mL, 1:2000; Chemicon), or biotin rat anti-mouse Cd45 (0.5 mg/mL; 1:800; BD Bioscience, San Jose, CA, USA) followed by incubation with the appropriate Alexa 546- or Alexa 555-conjugated goat secondary antibody (4 µg/mL, Life Sciences, Hercules, CA, USA). Vessels were stained with isolectin IB4-647 (IB-4; 1 mg/mL; Invitrogen, Carlsbad, CA). Cell nuclei were stained with Hoechst (1 mg/mL, Invitrogen, Carlsbad, CA, USA). For negative control staining, the primary antibodies were omitted, and no staining was observed. Immunofluorescence images were acquired by an IX81 microscope equipped with a confocal scan unit FV500 with 4 laser lines: Ar-Kr (488 nm), He-Ne red (646 nm), He-Ne green (532 nm) (Olympus, Tokyo, Japan), and a ultraviolet (UV) diode, using the scanning sequential mode to avoid bleed-through effects. Three-dimensional volumes were acquired over 10 µm stacks, with 0.23 µm step sizes. Imaris v.6 (Bitplane) and GNU Image Manipulation Program (GIMP) were used to obtain three-dimensional renderings of the images.

### 4.5. Statistical Analysis

Mice were randomly selected for surgery and were assigned across all cages and days. To minimize variability, all surgeries were performed by the same investigator. The results of the RT-qPCR analyses are presented as the normalized averages derived from the threshold cycle. For the tissue study (Figure 1, Figure 2, Figure 3, Figure 4 and Figure 9) and the primary cell culture study (Figure 10), the RT-qPCR and western blot results are presented as fold changes relative to the control (sham group (Figure 1, Figure 2, Figure 3, Figure 4 and Figure 9); unstimulated cells (Figure 10)). To determine the particular time points x TBI interaction (Figure 1, Figure 2, Figure 3, Figure 4 and Figure 9), ex vivo results (mean ± SEM) were evaluated using two-way ANOVA followed by Bonferroni’s multiple comparisons post hoc test. Additionally, the in vitro results (mean ± SEM) were evaluated using a t-test (Figure 10). All statistical analyses were performed with GraphPad Prism ver. 8.1.1 (330) (GraphPad Software, Inc., San Diego, CA, USA).

## 5. Conclusions

Depending on the elapsed time and brain regions, different cell types, such as neutrophils, Cd8+ cells, astroglia, macrophages, and microglia, play an essential role after TBI. Importantly, our results indicate that initiators of complement system activation in the ipsilateral brain structures may be responsible for cellular activation and chemotaxis. Among the complement initiators, it appears that the C1q/C1r/C1s complex plays an especially important role after brain damage in all studied brain structures (cortex, striatum, thalamus, and hippocampus). The primary microglial cell culture experiments herein suggest that those cells may be largely responsible for the biosynthesis of the initiators of the classical pathway but contributes very little to the production of lectin pathway initiators. In our opinion, selective targeting of microglia/macrophages and/or the C1q complex and ficolin A may be an effective strategy for human TBI therapy and in other neuroinflammation-related diseases; however, additional studies are required to investigate this hypothesis.

## Figures and Tables

**Table 1:** The *mannose binding lectin A* (*MBL-A*) and *mannose binding lectin C* (*MBL-C*) mRNA expression in brain structures (cortex, striatum, thalamus, and hippocampus) of sham/TBI-injured mice at selected time points (24 h, 4 days, 7 days, 2 weeks, 5 weeks) was not detected.

Brain Areas	*MBL-A*	*MBL-C*
cortex	not detected	not detected
striatum	not detected	not detected
thalamus	not detected	not detected
hippocampus	not detected	not detected

## Data Availability

Data is contained within the article and supplementary material.

## Supplementary Materials

The following are available online at https://www.mdpi.com/1422-0067/22/1/45/s1. Western blots details are presented as supplementary material 1 (Supplementary 1).

## Abbreviations

Aβ	Amyloid beta
BBB	Blood-brain barrier
C1qa	Complement component 1, q subcomponent, alpha polypeptide
C1ra	Complement component 1, r subcomponent A
C1s1	Complement component 1, s subcomponent 1
C3, C5b-9, C3b, C4b, C5b, C3a, C5a	Complement components
CC-1+	The oligodendrocyte-specific antibody
cC1qR	The collagen-like tail of C1q
CCI	Controlled cortical impact
CCL2	C-C motif chemokine 2
CCL3	C-C motif chemokine 3
Cd177	Cluster of Differentiation 177
Cd45	Cluster of differentiation 45
Cd8	Cluster of differentiation 8
cDNA	Complementary DNA
CNS	Central nervous system
DNA	Deoxyribonucleic acid
ECL	Enhanced chemiluminescence
gC1qR	Globular head of the C1q
GFAP	Glial fibrillary acidic protein
GIMP	GNU Image Manipulation Program
Hprt	Hypoxanthine guanine phosphoribosyl transferase
IBA-1	Allograft inflammatory factor 1
IL-6	Interleukin 6
IL-beta	Interleukin 1 beta
LPS	Lipopolysaccharide
MBL	Mannose-binding lectin
MBL-A	Mannose binding lectin A
MBL-C	Mannose binding lectin C
MMPs	Matrix metalloproteinases
mRNA	Messenger RNA
NGF	Nerve growth factor
NGS	Normal goat serum
Olig2	Oligodendrocyte transcription factor 2
PBS	Phosphate-buffered saline
PVDF	Polyvinylidene fluoride
RIPA	Radioimmunoprecipitation assay buffer
ROS	Reactive oxygen species
RPM	Revolutions per minute
RT	Room temperature
RT-qPCR	Real-time quantitative polymerase chain reaction
SEM	Standard error of the mean
TBI	Traumatic brain injury
TBST	Tris-buffered saline with 0.1% Tween 20
TGF-β	Transforming growth factor β
TMEM119	Transmembrane protein 119
TNF-α	Tumor Necrosis Factor alpha
UV	Ultraviolet
