# Supplementary material for: Initiators of Classical and Lectin Complement Pathways Are Differently Engaged after Traumatic Brain Injury—Time-Dependent Changes in the Cortex, Striatum, Thalamus and Hippocampus in a Mouse Model
Source: Int J Mol Sci. 2020 Dec 22;22(1):45. doi: 10.3390/ijms22010045 (PMC7793095; doi:10.3390/ijms22010045)
Supplement: Supplementary file 1 [file ijms-22-00045-s001.pdf]

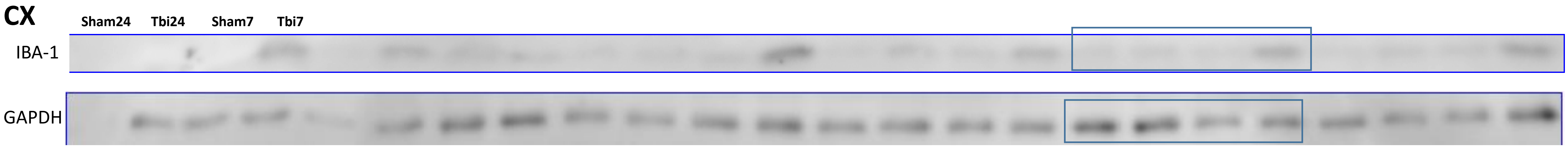
CX
Sham24
Tbi24
Sham7
Tbi7
IBA-1
GAPDH

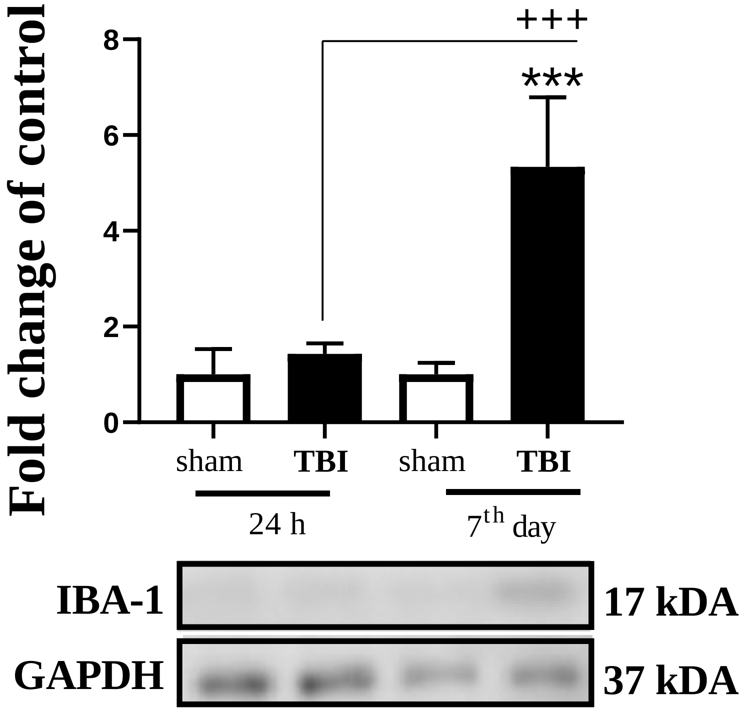
Fold change of control
8
6
4
2
0
+++
***
sham
TBI
sham
TBI
24 h
7th day
IBA-1
GAPDH
17 kDA
37 kDA

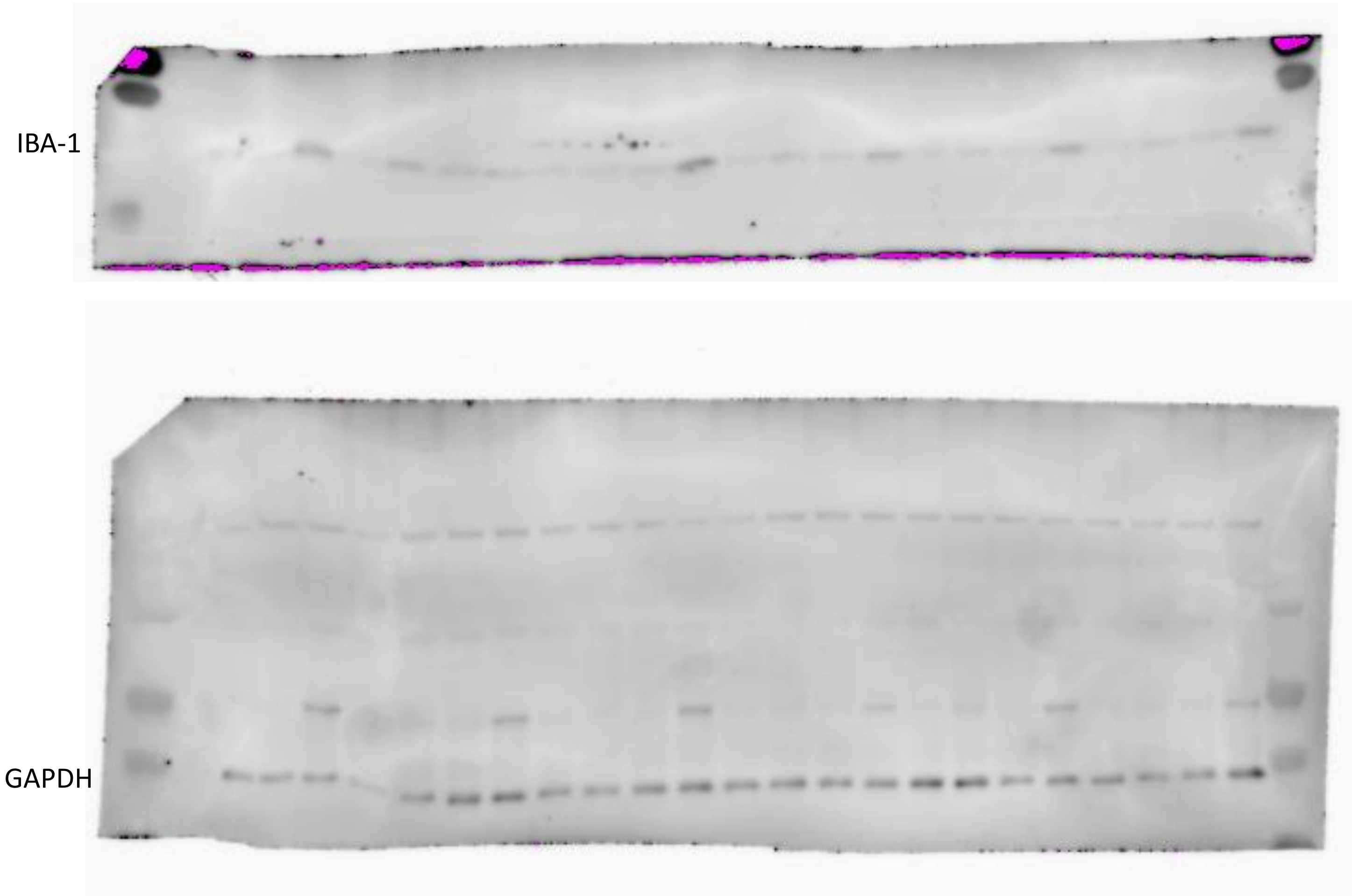
IBA-1
GAPDH

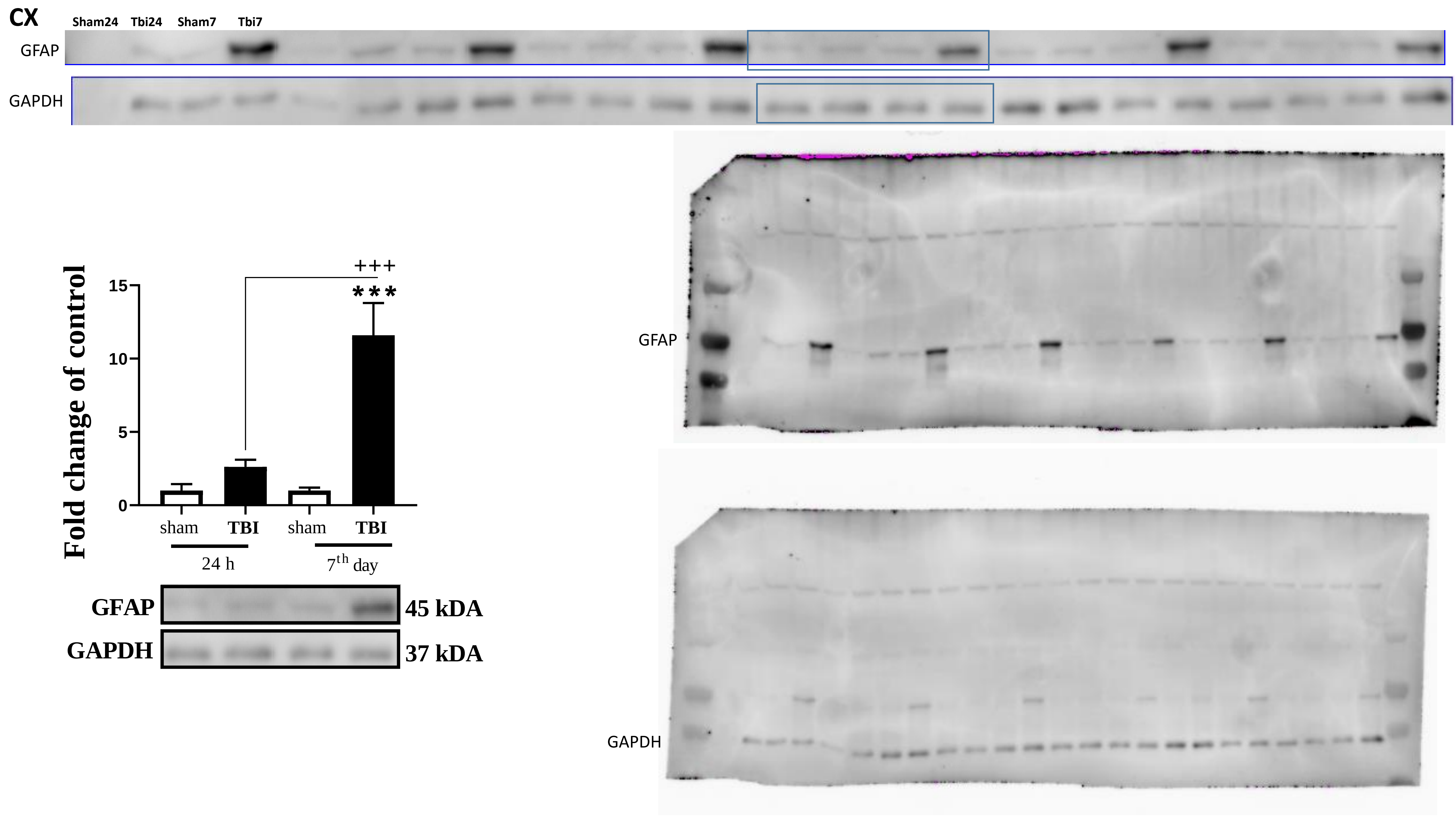
CX
Sham24
Tbi24
Sham7
Tbi7
GFAP
GAPDH
Fold change of control
15
10
5
0
+++
***
sham
TBI
sham
TBI
24 h
7th day
GFAP
45 kDA
GAPDH
37 kDA
GFAP
GAPDH

STR

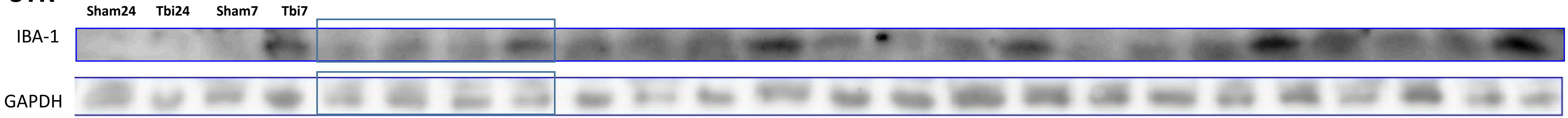

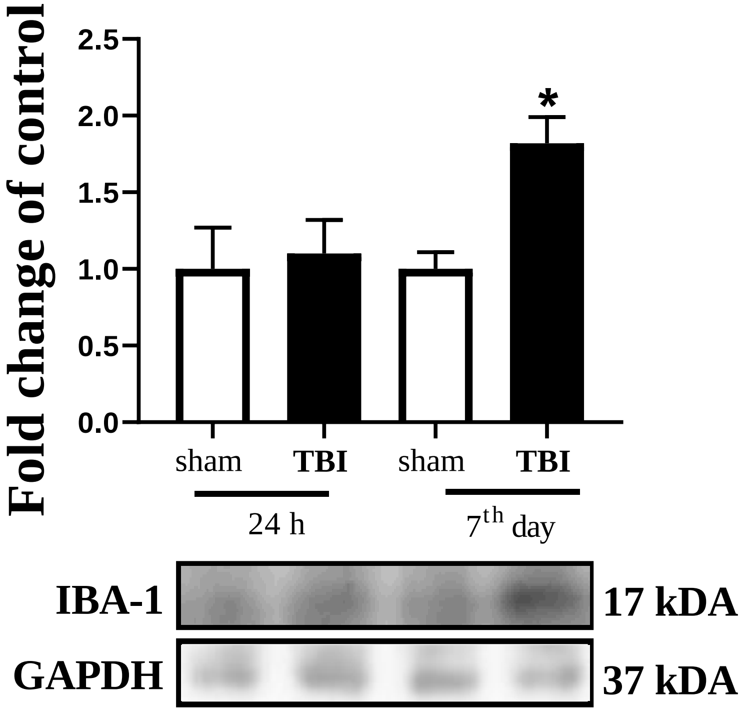

IBA-1

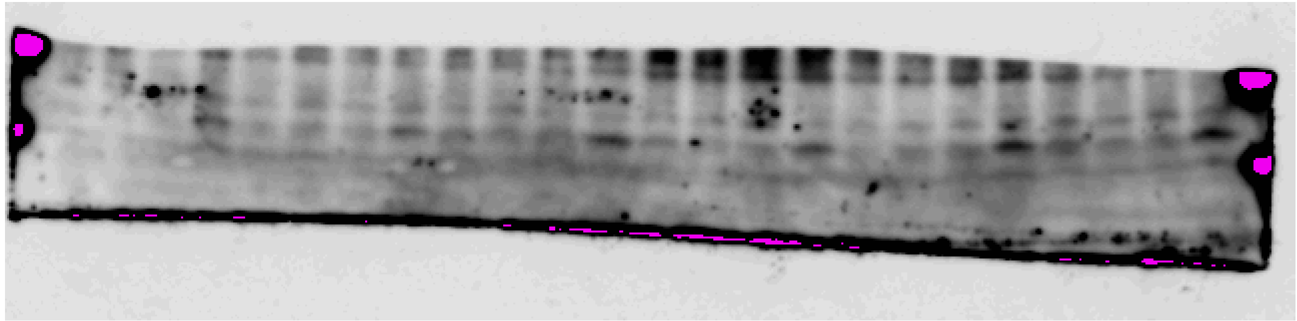

GAPDH

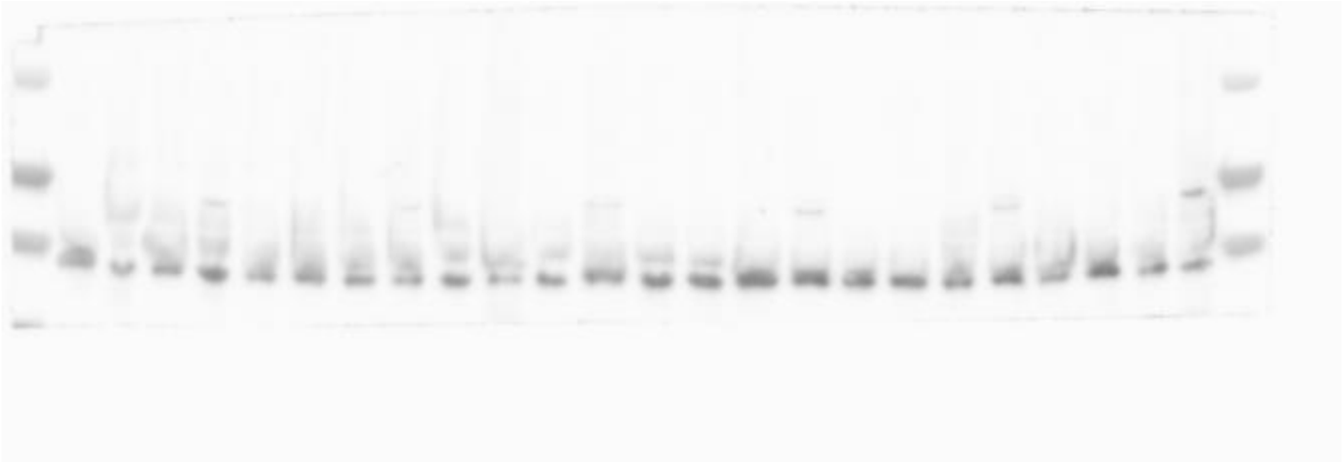

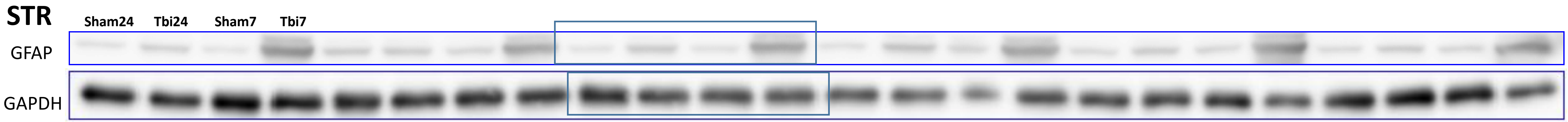
STR
Sham24
Tbi24
Sham7
Tbi7
GFAP
GAPDH

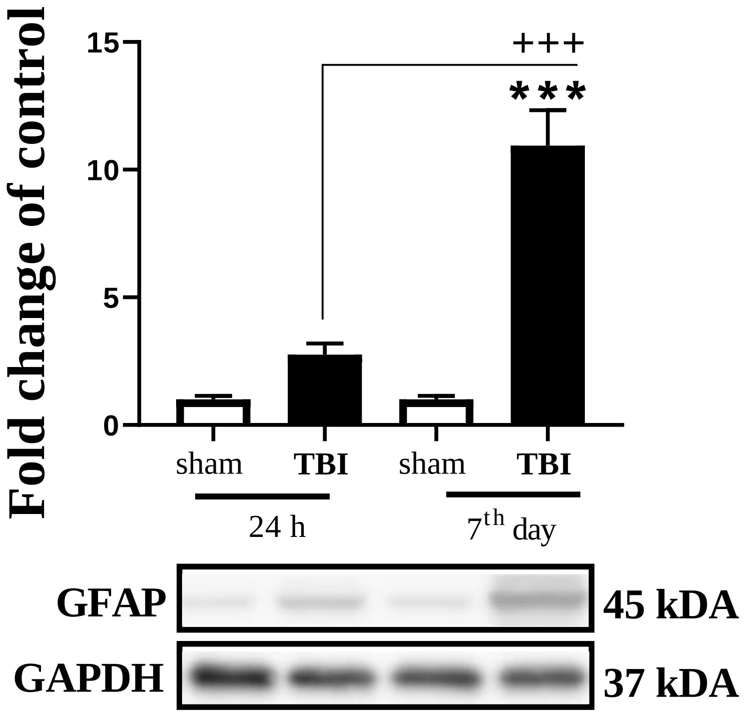
Fold change of control
15
10
5
0
+++
***
sham
TBI
sham
TBI
24 h
7th day
GFAP
45 kDA
GAPDH
37 kDA

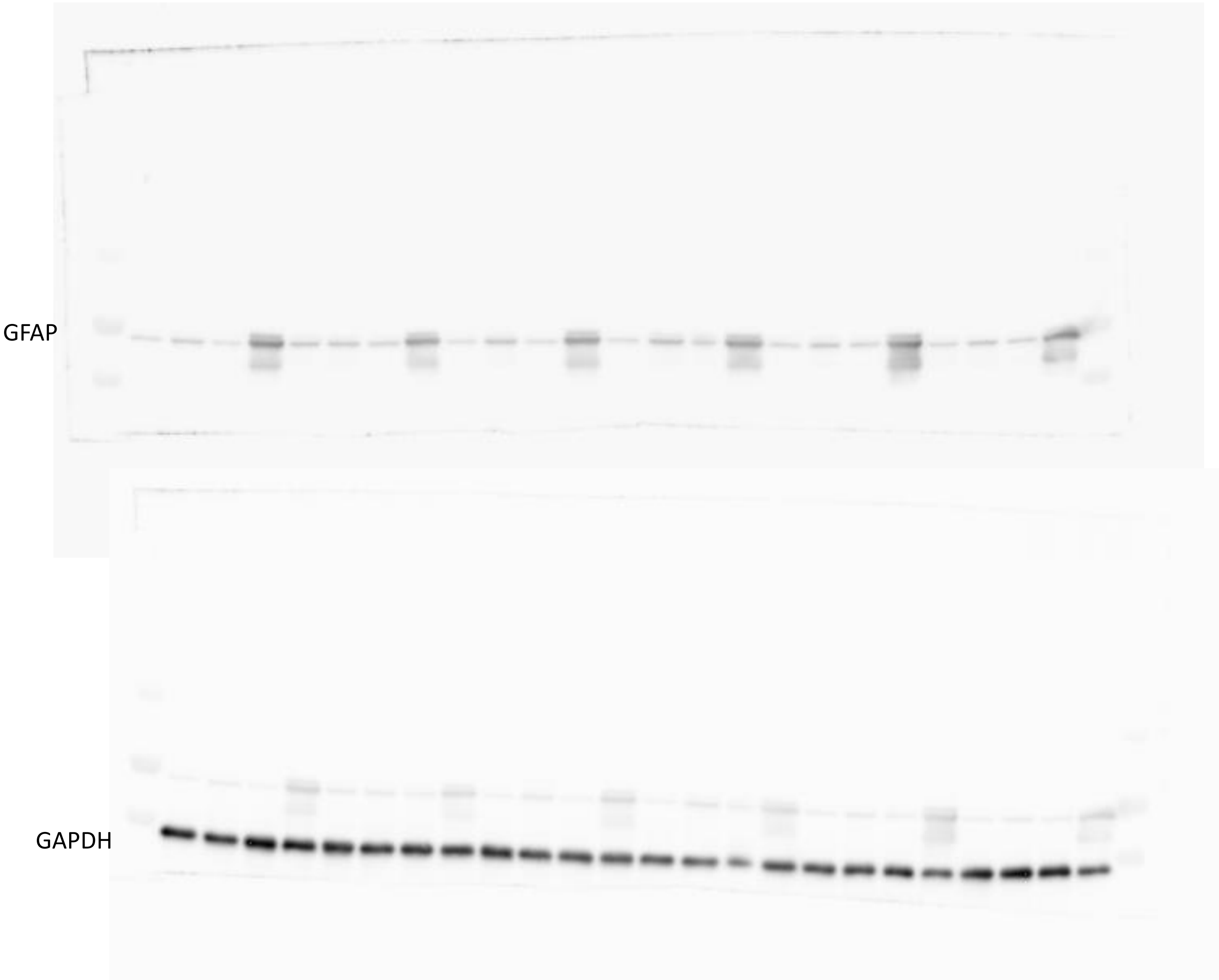
GFAP
GAPDH

TH

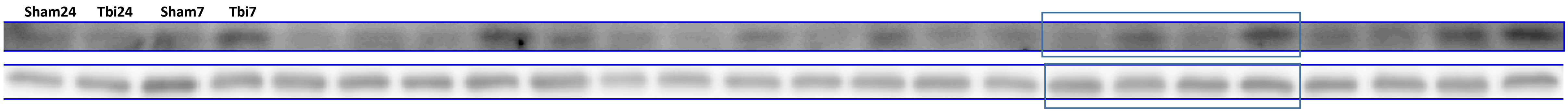

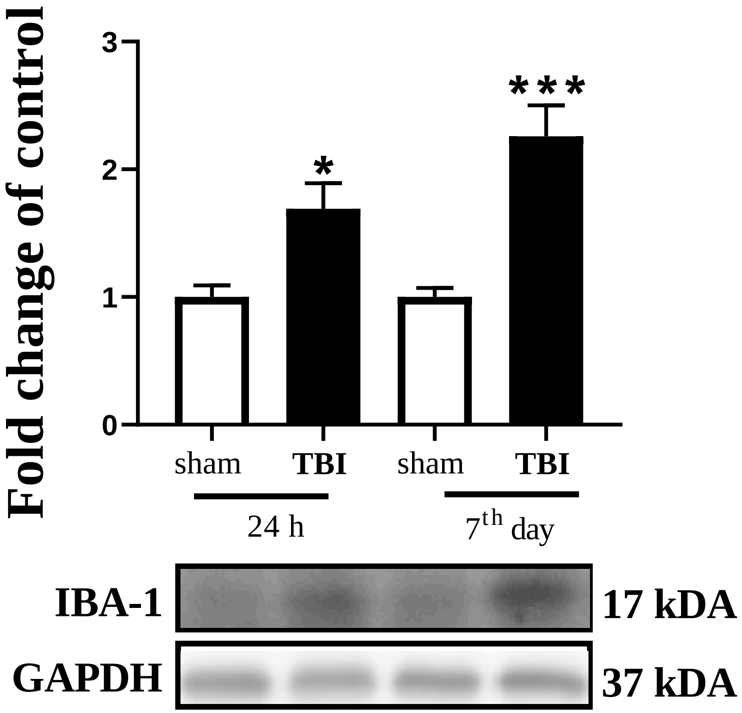

IBA-1

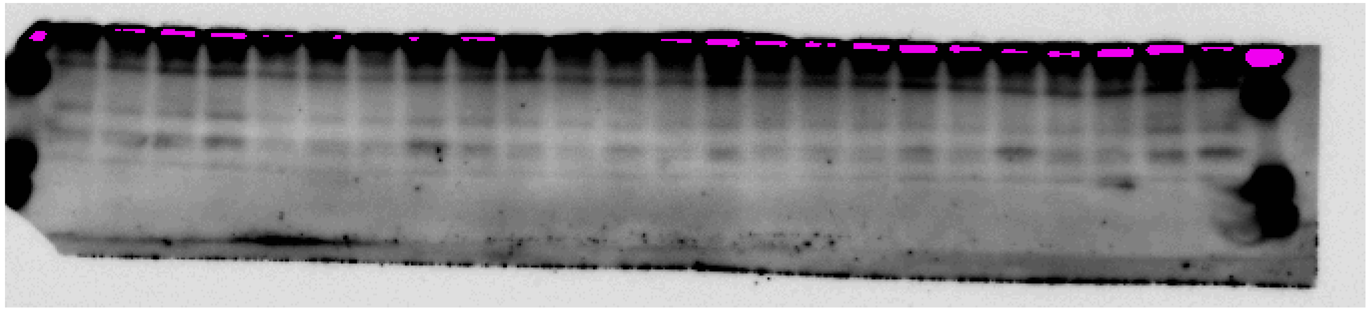

GAPDH

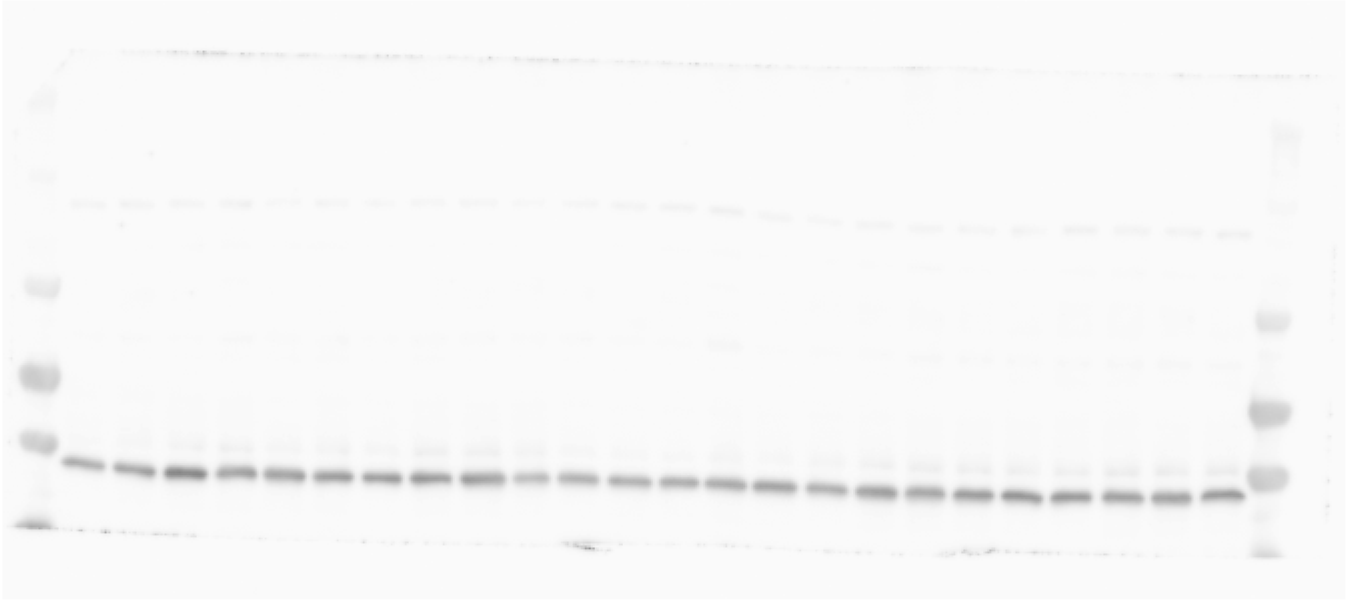

TH

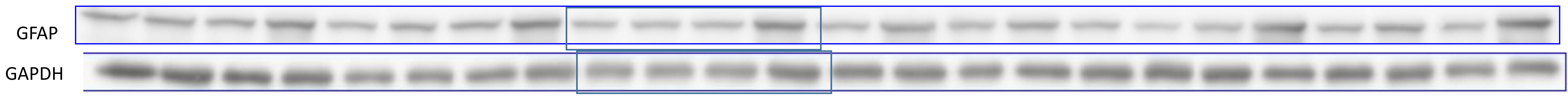

Sham24
Tbi24
Sham7
Tbi7
GFAP
GAPDH

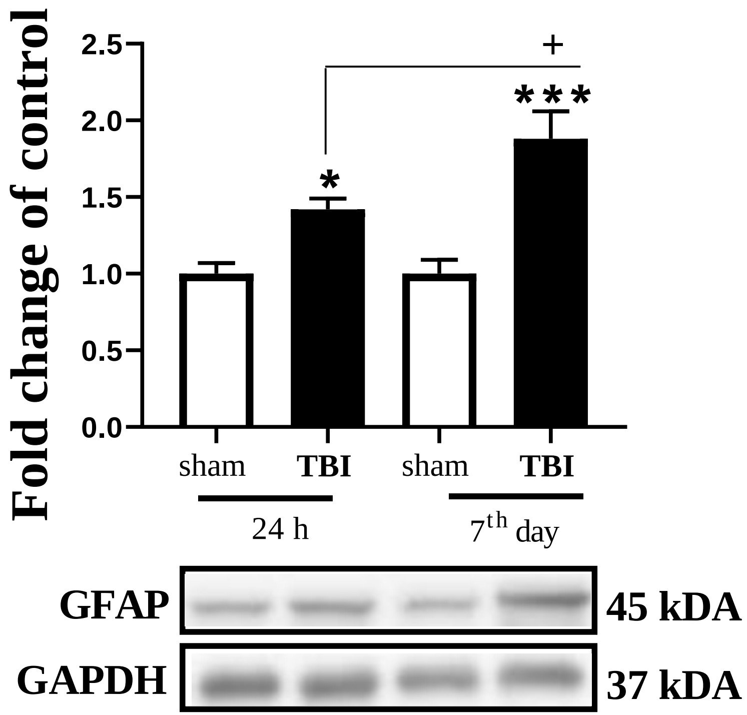

Fold change of control
2.5
2.0
1.5
1.0
0.5
0.0
+
***
*
sham
TBI
sham
TBI
24 h
7th day
GFAP
GAPDH
45 kDA
37 kDA

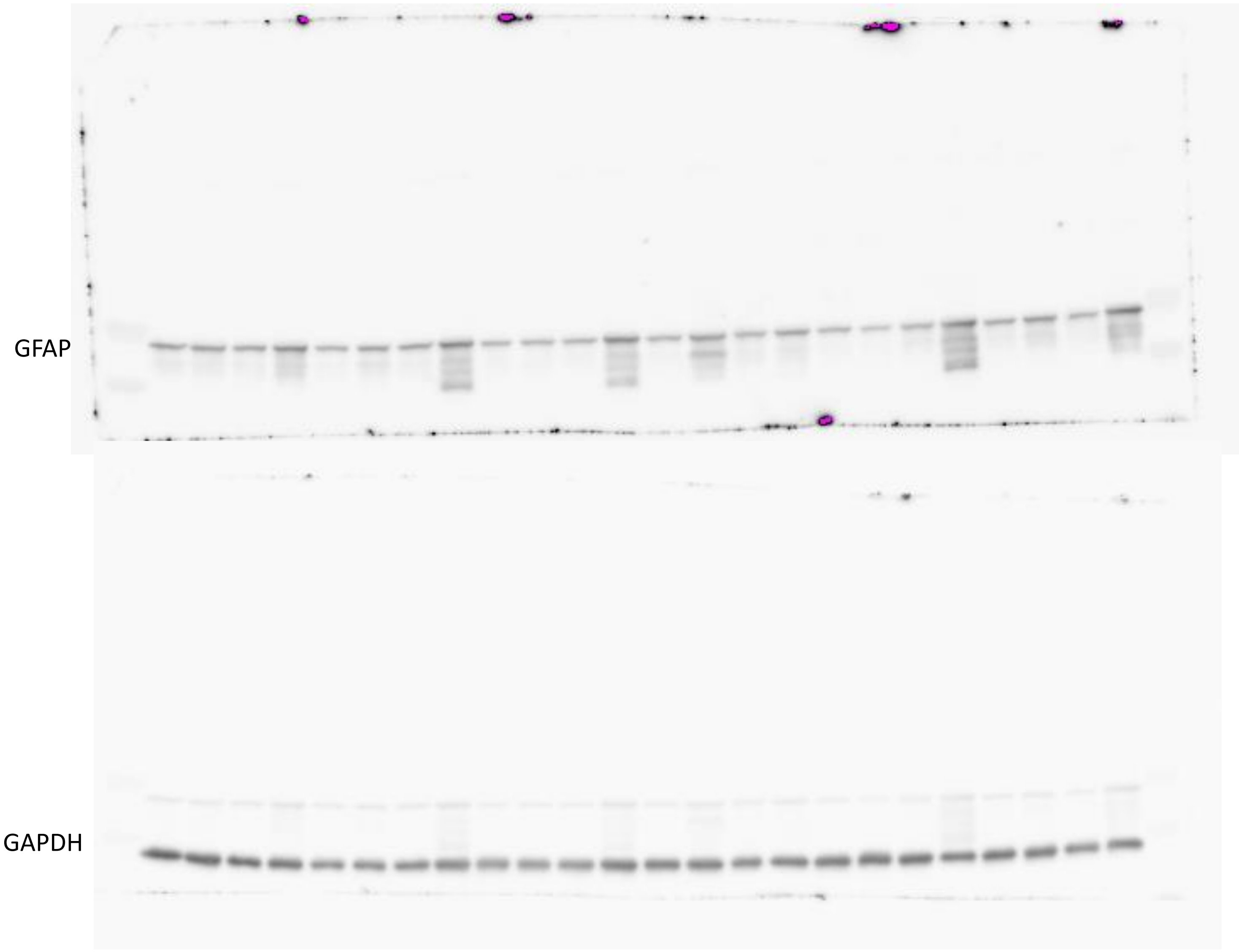

GFAP
GAPDH

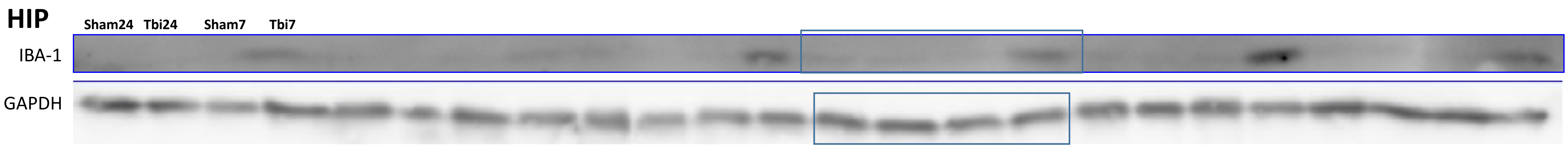
HIP
Sham24
Tbi24
Sham7
Tbi7
IBA-1
GAPDH

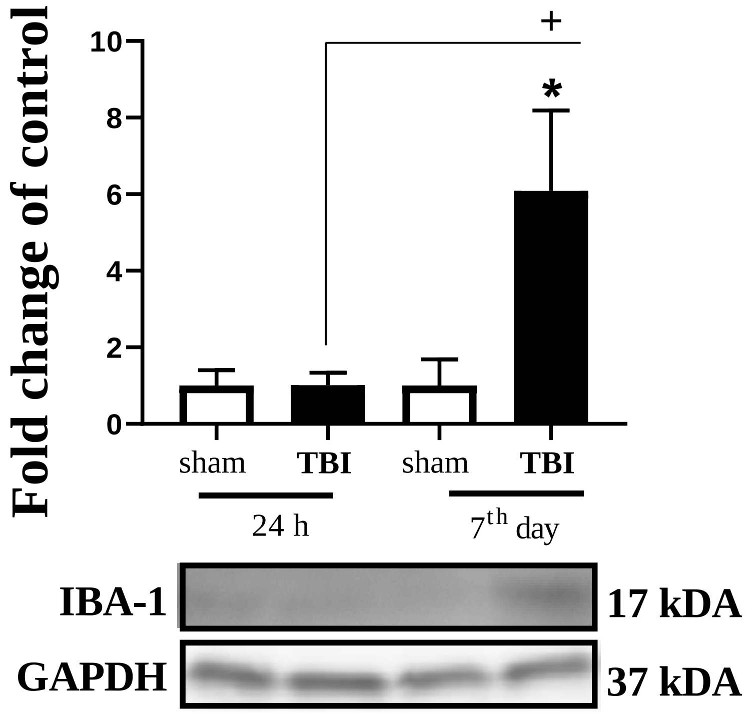
Fold change of control
10
8
6
4
2
0
+
*
sham
TBI
sham
TBI
24 h
7th day
IBA-1
17 kDA
GAPDH
37 kDA

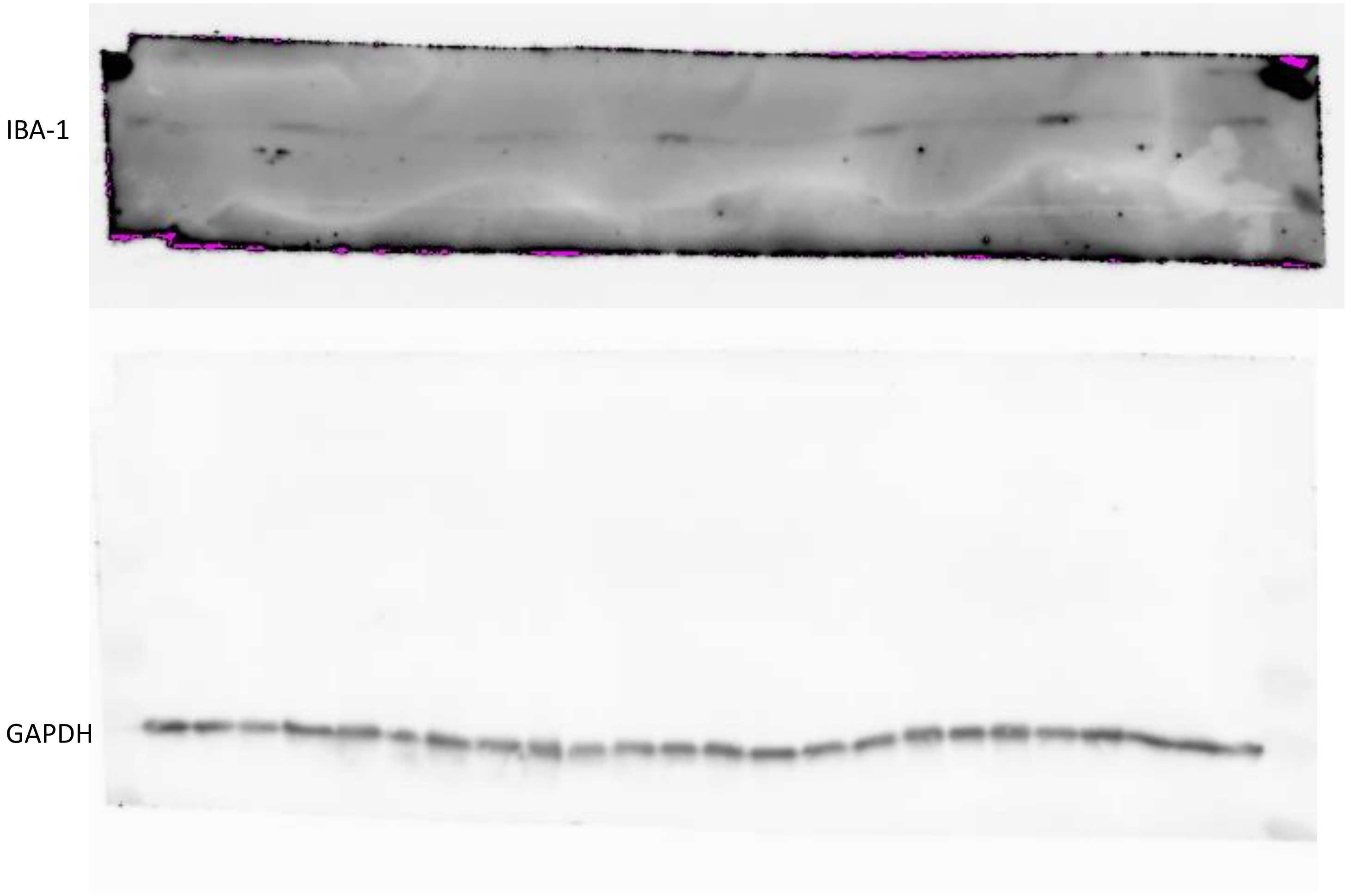
IBA-1
GAPDH

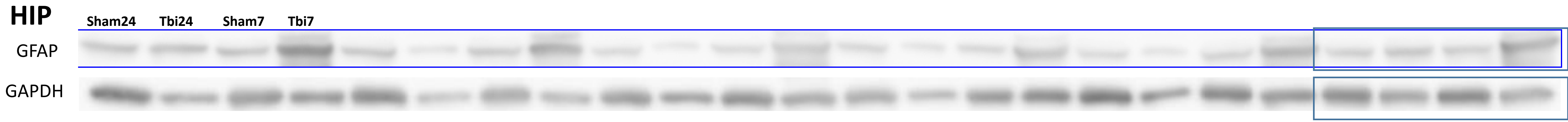
HIP
Sham24
Tbi24
Sham7
Tbi7
GFAP
GAPDH

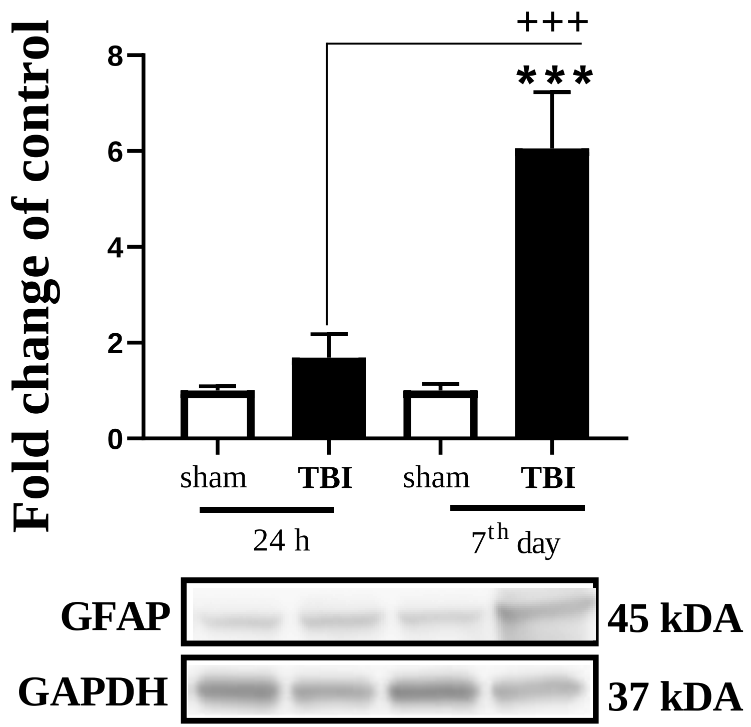
Fold change of control
+++
***
8
6
4
2
0
sham
TBI
sham
TBI
24 h
7th day
GFAP
45 kDA
GAPDH
37 kDA

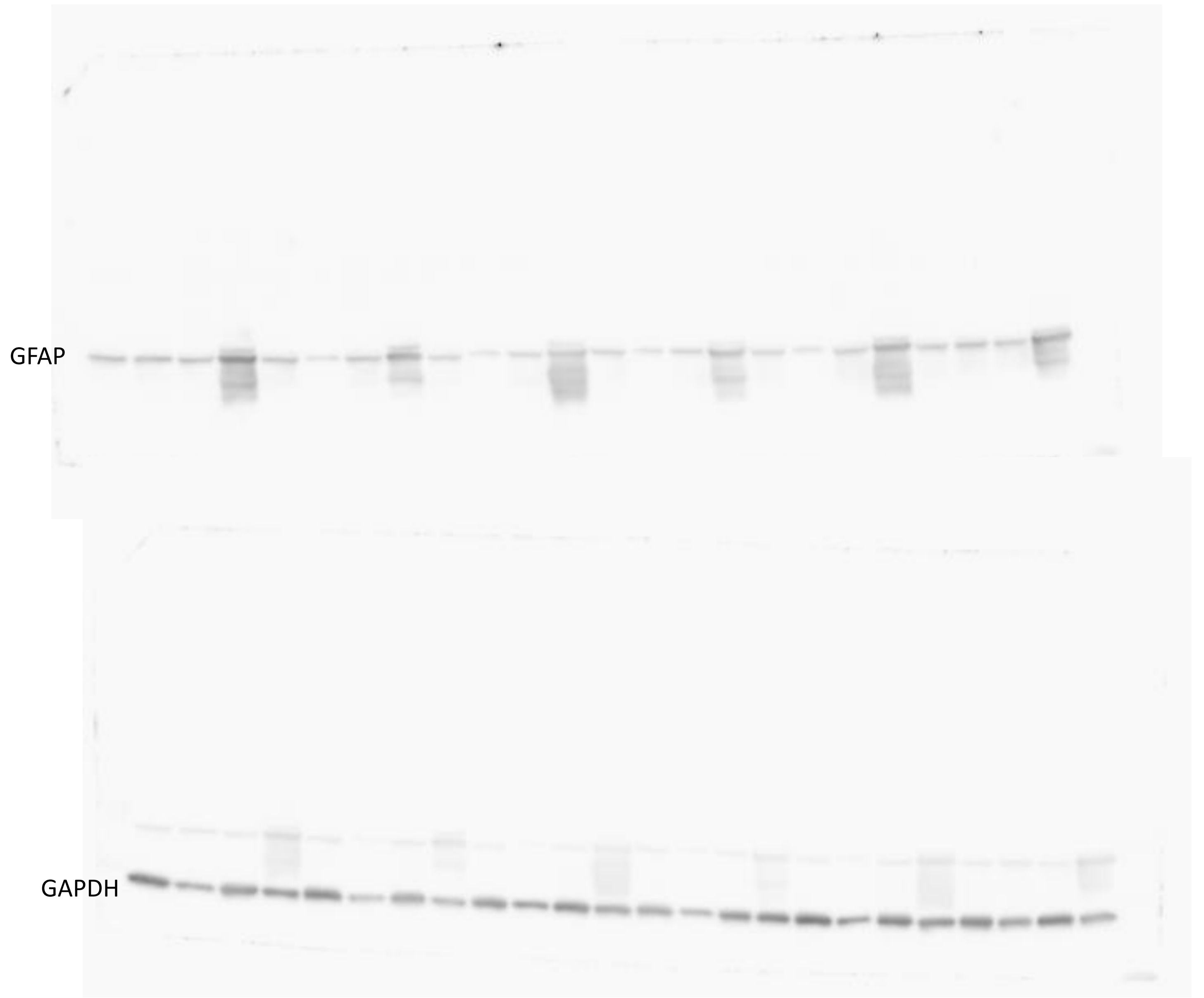
GFAP
GAPDH
